# Hourly future climate scenario datasets for impact assessment of climate change considering simultaneous interactions among multiple meteorological factors

**DOI:** 10.1016/j.dib.2022.108047

**Published:** 2022-03-11

**Authors:** Yuki Hiruta, Noriko N. Ishizaki, Shuichi Ashina, Kiyoshi Takahashi

**Affiliations:** a Social Systems Division, National Institute for Environmental Studies, 16-2 Onogawa, Tsukuba, Ibaraki 305-8506, Japan; b Center for Climate Change Adaptation, National Institute for Environmental Studies, 16-2 Onogawa, Tsukuba, Ibaraki 305-8506, Japan

**Keywords:** Future climate scenario, Historical weather data, Hourly time resolution, Global climate model (GCM), Representative concentration pathways (RCPs)

## Abstract

Assessing the impacts of climate change in multiple fields, such as energy, land and water resources, and human health and welfare is important to find effective strategies to adapt to a changing climate and to reduce greenhouse gases. Many phenomena influenced by climate change have diurnal fluctuations and are affected by simultaneous interactions among multiple meteorological factors. However, climate scenarios with detailed (at least hourly) resolutions are usually not available. To assess the impact of climate change on such phenomena while considering simultaneous interactions (e.g., synergies), climate scenarios with hourly fluctuations are indispensable. However, because meteorological indicators are not independent, the value of one indicator varies as a function of other indicators. Therefore, it is almost impossible to determine the functions that show all relationships among meteorological elements considering the geographical and temporal (both seasonal and time of a day) characteristics. Therefore, generating hourly scenarios that include possible combinations of meteorological indicators for each hourly observation unit is a challenging problem. In this study, we provide secondary future climate scenario datasets that have hourly fluctuations with reasonable combinations of meteorological indicator values that are likely to occur simultaneously, without losing the long-term climate change trend in the existing daily climate scenarios based on global climate models. Historical hourly weather datasets observed from 2017 to 2019 (the reference years) are used to retrieve short-term fluctuations. Bias-corrected daily future climate scenario datasets generated using four global climate models (GFDL CM3, HadGEM2-ES, MIROC5, and MRI-CGCM3) and two Representative Concentration Pathways (RCP8.5 and 2.6) are used to model long-term climate change. A total of 48 different types of hourly future scenario datasets for five meteorological indicators (temperature, solar radiation, humidity, rainfall, and wind speed) were acquired, targeting a projection period from 2020 to 2080, for 10 weather stations in Japan. The generated hourly climate scenario datasets can be used to project the quantitative impacts of climate change on targeted phenomena considering simultaneous interactions among multiple meteorological factors.

## Specifications Table

**Table:** 

Subject	Renewable energy, sustainability and the environment
Specific subject area	Impact assessment of climate change
Type of data	Table
How data were acquired	The data were acquired via statistical computing using R (a free software environment for statistical computing and graphics).
Data format	Raw and analyzed
Description of data collection	The hourly future climate scenarios are provided as secondary data that are generated by adding short-term fluctuations obtained from hourly historical weather data [1] to daily future climate scenarios [2,3].
Data source location	Cities: Sapporo, Sendai, Tokyo, Nagoya, Kanazawa, Osaka, Hiroshima, Matsuyama, Fukuoka, and Naha Country: Japan Latitude and longitude: (See Table 1) Primary data sources: **Hourly historical weather data:** Japan Meteorological Agency, Historical Weather Data, (2020). http://www.data.jma.go.jp/obd/stats/etrn/index.php (accessed January 20, 2020) [1]. **Daily future climate scenario datasets:** Ishizaki NN. Bias corrected climate scenarios over Japan based on CDFDM method using CMIP5 2019. https://doi.org/10.17595/20200415.001[2]. A-PLAT Pro. Cent Clim Chang Adapt Natl Inst Environ Stud. https://ccca-scenario.nies.go.jp/data/jpn_cdfdm/nies2019[3].
Data accessibility	Data is hosted on Mendeley Data [4]. Repository name: Mendeley Data Data identification number: doi:https://doi.org/10.17632/h8wtdrnb25.1 Direct URL to data: https://doi.org/10.17632/h8wtdrnb25.1
Related research article	Y. Hiruta, N.N. Ishizaki, S. Ashina, K. Takahashi, Regional and temporal variations in the impacts of future climate change on Japanese electricity demand: Simultaneous interactions among multiple factors considered, Energy Convers. Manag. X. (2022) 100172. https://doi.org/10.1016/j.ecmx.2021.100172[5].

## Value of the Data

• Many phenomena influenced by climate change have diurnal fluctuations and are affected by simultaneous interactions among multiple meteorological factors. However, climate scenarios with detailed (at least hourly) resolutions are not usually available. To assess the impact of climate change on such phenomena considering simultaneous interactions (e.g., synergies), we need climate scenarios with hourly fluctuations that have reasonable combinations of meteorological indicator values that are likely to occur simultaneously.
• Assessing the impacts of climate change is important in many fields to find effective strategies to adapt to a changing climate and reduce greenhouse gases. Researchers and practicians who assess the impact of climate change on phenomena with diurnal variations in fields such as energy, agriculture, land and water resources, human health and welfare, and biological diversity can benefit from the datasets.
• The hourly climate scenario datasets can be used in models to project the quantitative impacts of climate change on targeted phenomena considering simultaneous interactions among multiple factors.
• Meteorological indicators are not independent; they vary as a function of other indicators, such as the relationship between temperature and relative humidity presented in a psychrometric chart. It is almost impossible to determine the functions that show all relationships among meteorological elements considering geographical and temporal (both seasonal and time of a day) characteristics. The generated hourly climate scenario datasets have hourly fluctuations that have actually been observed while maintaining the long-term climate change trend of the existing daily climate scenarios based on global climate models.
• The proposed method to generate hourly future climate scenarios is applicable to data observed at other Japanese weather stations as well as those in other nations.

## 1 Data Description

The hourly future climate scenario datasets (h-CSs) consist of a total of 480 CSV files divided into 16 folders. The 480 CSV files comprise combinations of two types of data, four global climate models (GCMs), two Representative Concentration Pathways (RCPs), three reference years, and 10 weather stations in Japan. A list of the folder and file names is in Appendix A. The following is an example of how the folders and files are named.

Folder name: h-CS_CN_MIROC5_rcp26.

File name: h-CS_CN_MIROC5_rcp26_2017_06_Osaka.csv.

The first term (h-CS in this example) of the folder and file names indicates the abbreviated name of the future climate scenario datasets provided. The second term (CN) indicates the data type: OR (original) or CN (climate neutral). The third term (MIROC5) indicates the type of GCM used to project future climate scenarios. Four types of GCMs (MIROC5, MRI-CGCM3, GFDL CM3, and HadGEM2-ES) were used to generate the hourly future climate scenario datasets. The fourth term (rcp85) indicates the RCP used to project future climate scenarios. RCP8.5 and RCP2.6 were used to generate the hourly future climate scenario datasets. The fifth term (2017) in the file name shows the reference year, which denotes the year in which the hourly historical weather being used to add hourly fluctuations to the daily future climate scenarios were observed. The sixth (06) and seventh (Osaka) terms show the identification number and name of the weather station for which the h-CS was constructed, respectively.

## 2 Experimental Design, Materials and Methods

**Fig. 1:**
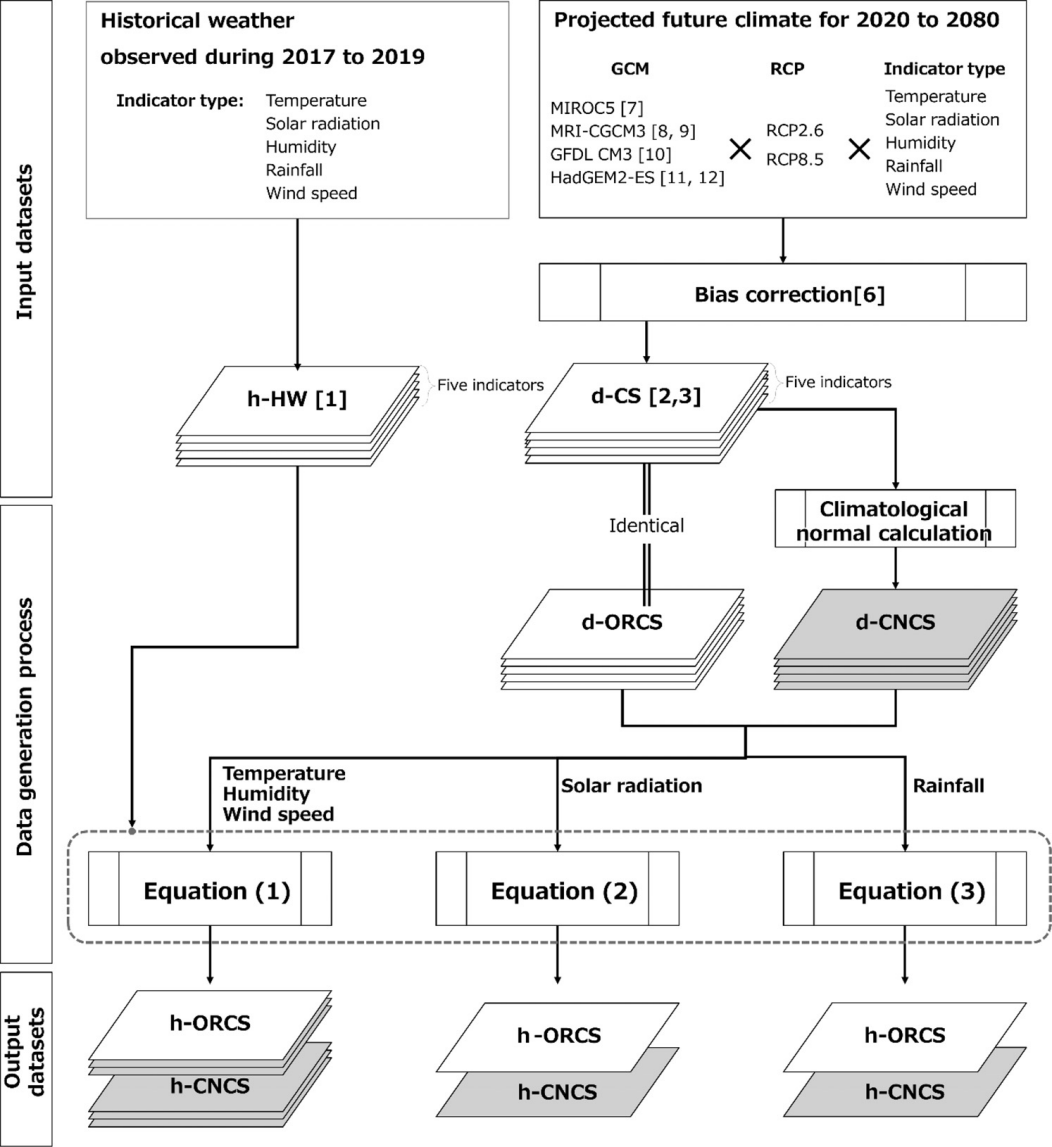
The framework of the h-CS generation process. h-HW, hourly historical weather; d-CS, daily climate scenario; d-ORCS, daily (original) climate scenario; d-CNCS, daily climate scenario for climatological normal; h-ORCS, hourly (original) climate scenario; h-CNCS, hourly climate scenario for climatological normal; RCP, Representative Concentration Pathways; GCM, global climate models.

**Fig. 2:**
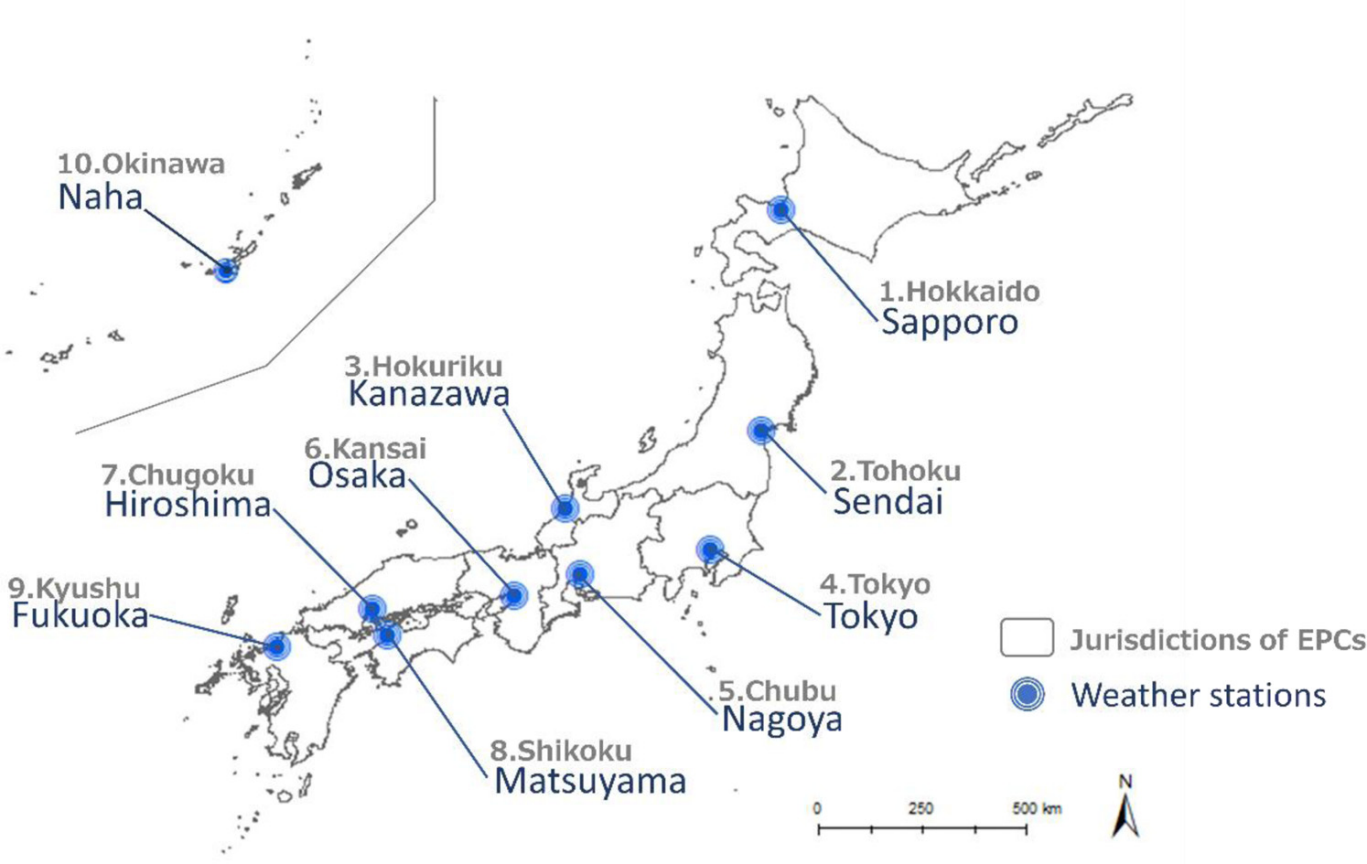
The location of the targeted weather stations [1] and the jurisdictions of 10 EPCs [5].

Fig. 1 illustrates the framework of the h-CS generation process. The h-CSs for five meteorological indicators (temperature, solar radiation, humidity, rainfall, and wind speed) were generated for 10 weather stations located in the most populated cities in Japan (Sapporo, Sendai, Tokyo, Nagoya, Kanazawa, Osaka, Hiroshima, Matsuyama, Fukuoka, and Naha) in each jurisdiction of 10 major electric power companies (EPCs) from Hokkaido to Okinawa (Table 1, Fig. 2). The h-CSs were generated by adding the short-term fluctuations obtained from hourly historical weather data (h-HW) [1] to the daily future climate scenario (d-CS) [2,3]. The h-HWs were used as reference data for short-term fluctuations presenting reasonable combinations of meteorological factors that would likely occur simultaneously, whereas d-CSs were used to present long-term climate change projected by the GCMs.

**Table 1:** Latitude and longitude of the weather stations [1] and the jurisdictions of 10 EPCs [5].

Weather Stations	EPCs	Latitude		Longitude
Sapporo	1. Hokkaido	43° 03′ 36′' N		141° 19′ 42′' E
Sendai	2. Tohoku	38° 15′ 42′' N		140° 53′ 48′' E
Kanazawa	3. Hokuriku	36° 35′ 18′' N		136° 38′ 00′' E
Tokyo	4. Tokyo	35° 41′ 30′' N		139° 45′ 00′' E
Nagoya	5. Chubu	35° 10′ 00′' N		136° 57′ 54′' E
Osaka	6. Kansai	34° 40′ 54′' N		135° 31′ 06′' E
Hiroshima	7. Chugoku	34° 23′ 54′' N		132° 27′ 42′' E
Matsuyama	8. Shikoku	33° 50′ 36′' N		132° 46′ 36′' E
Fukuoka	9. Kyushu	33° 34′ 54′' N		130° 22′ 30′' E
Naha	10. Okinawa	26° 12′ 24′' N		127° 41′ 12′' E

### 2.1 Future climate scenarios

The d-CSs from 2020 to 2080 (the projection period) were used to indicate long-term future climate change. The d-CSs [2,3] have a resolution of 1 km and are the bias-corrected [6] future climate scenario datasets generated by the four GCMs (MIROC5 [7], MRI-CGCM3 [8,9], GFDL CM3 [10], and HadGEM2-ES [11,12]) for the two RCPs (8.5 and 2.6) for five meteorological indicators. Table 2 summarizes the details of the four GCMs. The datasets were organized by two research programs (the Climate Change Adaptation Program [13] and the related former program, S-8 Comprehensive Study on Impact Assessment and Adaptation for Climate Change (2010–2014) [14]) and were provided by A-PLAT Pro [2,3].

**Table 2:** Climate models used in this study [5].

Model	Centers	Ref.
**MIROC5**	Atmosphere and Ocean Research Institute (The University of Tokyo), National Institute for Environmental Studies, and Japan Agency for Marine-Earth Science and Technology Japan	[7]
**MRI-CGCM3**	Meteorological Research Institute Japan	[8,9]
**GFDL CM3**	Geophysical Fluid Dynamics Laboratory USA	[10]
**HadGEM2-ES**	Met Office Hadley Centre UK	[11], [12]

To detect the possible range of quantitative impacts of climate change on the targeted phenomena using the generated hourly scenarios, we selected the four GCMs from among those available in the Coupled Model Intercomparison Project Phase 5 (CMIP5) to cover a wide range (maximum, upper one-third, lower one-third, and minimum) of projected temperatures and precipitation levels based on the analysis conducted by Hanasaki et al [15]. RCP8.5 and RCP2.6 were selected because they represent the two extreme pathways of radiative forcing values for the year 2100, 8.5 and 2.6 W/m^2^, respectively, of the four RCPs for climate modeling and research for the IPCC fifth Assessment Report (AR5).

Two types of d-CSs were generated for the five meteorological indicators. The first type, OR, comprised the original climate scenario dataset. The second, CN, is the climatological normal calculated from the OR, indicating the climate that would not be affected by shorter-term meteorological phenomena. The d-CSs for OR and CN were expressed as d-ORCS and d-CNCS, respectively, and h-ORCS and h-CNCS are the hourly future climate scenario datasets for OR and CN. d-CNCS was defined as a 31-year (including the target year and the previous and subsequent 15 years from it) average of each meteorological indicator for each day. This is not exactly the same as “climatological standard normal” defined by the World Meteorological Organization (WMO) [16,17], which represents the averages of climatological data computed for the following consecutive periods of 30 years. In this data generation, the previous and subsequent 15 years were included to generate a climatological normal that is likely to be experienced on that year on the day.

### 2.2 Observed meteorological data

Hourly historical weather datasets (h-HWs) [1] for five meteorological indicators (Table 3) were used as reference data for short-term fluctuations presenting reasonable combinations of meteorological factors that were likely to occur simultaneously. We used h-HWs observed from 2017 to 2019 (i.e., the reference years) based on the assumptions that the 26,280 hours observations during these three years (24 h × 365 days × 3 reference years) provide enough variations to consider the possible combinations of five meteorological elements. The solar radiation (SUN) data at the Kanazawa weather station were missing throughout the reference years; therefore, data from observations recorded at the Fukui station, which is also located in the Hokuriku region, were used instead.

**Table 3:** Meteorological indicators.

Meteorological Indicator	Unit	Data Description
Temperature (TEMP)	°C	Hourly average air temperature
Solar radiation (SUN)	MJ/m^2^	Hourly total solar radiation
Humidity (HUM)	%	Hourly average relative humidity
Rainfall (RAIN)	mm	Hourly total rainfall
Wind speed (WIND)	m/s	Average wind speed during the 10 min before each hour

### 2.3 Generation of hourly climate scenario datasets

The h-CSs were generated by adding the short-term fluctuations obtained from hourly historical weather data [1] (h-HW) into the daily future climate scenario [6] (d-CS). To generate reasonable combinations of meteorological indicators that would likely be observed simultaneously at each hour for the hourly climate scenario datasets (h-CSs), we used the hourly data that were actually observed in the three reference years to provide hourly fluctuations for the d-CSs. Therefore, three different h-CSs based on the reference year used were generated for each d-CS.

In accordance with the aggregation method generally used to convert meteorological data observed at ground stations to daily values [18], we used three types of methods to generate h-CSs. For the meteorological elements that are aggregated using mean value (TEMP, HUM, and WIND), Eq. (1) was used. For SUN, which is aggregated using the sum of the values and has a diurnal cycle, Eq. (2) was used. For RAIN, which is aggregated using the sum of the values and is also intermittent on a daily basis (i.e., rainfall is usually not observed every day), Eq. (3) was used. A, B, and C represent the type of meteorological elements that correspond to the three aggregation methods noted for Eqs. (1), (2), and (3), respectively.

(1) $$\left(h-CS_{A}\right)=\left(d-\text{maC}S_{A}\right)-\left(d-\text{maH}W_{A}\right)+\left(h-HW_{A}\right)$$

(2) $$\left(h-CS_{B}\right)=\left(h-HW_{B}\right)\times \left(d-CS_{B}\right)/\left(d-\text{sumH}W_{B}\right)$$

(3) $$\left(h-CS_{C}\right)=\left(h-HW_{C}\right)\times \left(m-\text{sumC}S_{C}\right)/\left(m-\text{sumH}W_{C}\right)$$

h−CS is the hourly future climate scenario, d−maCS is the moving average of the daily future climate scenarios, d−maHW is the moving average of observed daily historical weather of the reference years, h−HW is the observed hourly historical weather of the reference years, d−CS is the daily future climate scenario, d−sumHW is the daily total of the observed hourly historical weather of the reference years, m$-\text{sumC}S_{C}$ is the monthly total of the daily future climate scenario, and m−sumHW is the monthly total of the observed hourly historical weather of the reference years.

For $h-CS_{A}$, the moving averages ($d-\text{maC}S_{A}$ and $d-\text{maH}W_{A}$) were calculated for both daily future climate scenarios ($d-CS_{A}$) and observed daily historical weather data ($d-HW_{A}$). The value of $h-CS_{A}$ was then obtained by Eq. (1), which stems from the idea of adding hourly fluctuations ($\left(h-HW_{A}\right)-\left(d-\text{maH}W_{A}\right)$) to the mid-term trend of the future climate ($d-\text{maC}S_{A}$). For $d-\text{maC}S_{A}$ and $d-\text{maH}W_{A}$, an n-day moving average that included the previous and subsequent k days and the target day, was applied m times. Values of k=15 (31 days) and m=3 were selected. It was not possible to avoid some amount of arbitrariness in parameter selection. A value of m=3 was selected because it is used by the Japan Meteorological Agency to calculate the climatological normal in Japan. A value of k=15 was selected from several candidate parameters (5, 10,15, 20, 25, 30) by comparing the visualized moving averages in terms of showing an appropriately smooth sequence.

Eq. (2) for the $h-CS_{B}\;$of SUN stems from the idea that the proportions of each hourly future climate scenario ($h-CS_{B}$) relative to the daily future climate scenarios ($d-CS_{B}$) in a given day of the year should be the same as those of the hourly historical SUN ($h-HW_{B}$) relative to the daily aggregated hourly historical SUN(d−sum$HW_{B}$). Furthermore, the generated hourly future climate scenarios ($h-CS_{B}$) should keep the same values as the values in the original daily scenarios ($d-CS_{B}$) when they are aggregated to daily data.

Eq. (3) for $h-CS_{C}\;$of RAIN stems from the idea that the proportions of each hourly future climate scenario ($h-CS_{B}$) relative to monthly future climate scenarios ($m-\text{sumC}S_{C}$) should be the same as those of the hourly historical RAIN ($h-HW_{B}$) relative to the monthly aggregated hourly historical RAIN ($m-\text{sumH}W_{C}$). In addition, the generated hourly future climate scenarios ($h-CS_{B}$) should keep the same values as the values in the monthly aggregated original daily scenarios ($m-\text{sumC}S_{C}$) when they are aggregated to monthly data.

### 2.4 Attributes of the generated h–CSs

In total, 48 types of hourly future climate scenarios were obtained using the four GCMs, two RCPs, two data types, and three reference years. Therefore, 48 types of h−CS were derived for each hour unit from 2020 to 2080 for the 10 weather stations and the five meteorological indicators (Table 4).

**Table 4:** Attributes of generated h-CSs.

Data Types (2)	GCMs (4)	RCPs (2)	Reference Years (3)	Weather Stations (10)	Meteorological Indicators (5)
OR CN	MIROC5 MRI-CGCM3 GFDL CM3 HadGEM2-ES	RCP8.5 RCP2.6	2017 2018 2019	Sapporo Sendai Kanazawa Tokyo Nagoya Osaka Hiroshima Matsuyama Fukuoka Naha	Temperature (TEMP) Solar radiation (SUN) Humidity (HUM) Rainfall (RAIN) Wind speed (WIND)

### 2.5 Limitations

Some limitations should be noted. We used historical weather data (reference data) observed during 2017–2019 (i.e., the reference years) for short-term fluctuations. We applied the reference data based on two assumptions: (1) the 26,280 observations during the three reference years provide enough variations to consider the possible combinations of five meteorological elements, and (2) the same hourly fluctuations as those observed in the reference years will occur during the projection period (2020–2080).

When the generated hourly climate scenarios are applied to assess the impact of climate change, it is critical to make sure the aggregated assessed quantitative impacts of climate change on the targeted phenomena do not differ based on the reference year used. If the assessed quantitative impacts depend too greatly on the selected reference year, we need to consider more diverse reference years to understand the general insight about the quantitative impacts of climate change on the targeted phenomena. In addition, the hourly fluctuations may be enhanced in accordance with long-term climate change, and the daily scenarios also include uncertainties. We still need to develop ways to project long-term changes in hourly fluctuations that have reasonable simultaneous combinations among multiple meteorological factors in future climate scenarios.

Validation by comparing the distribution of the developed hourly scenario data and that of actual hourly observations made in the future remains as a future task to better understand changes in short-term fluctuations caused by long-term climate change.

## CRediT authorship contribution statement

**Yuki Hiruta:** Conceptualization, Methodology, Data curation, Visualization, Writing – original draft. **Noriko N. Ishizaki:** Conceptualization, Methodology, Data curation, Writing – review & editing, Supervision. **Shuichi Ashina:** Writing – review & editing, Supervision. **Kiyoshi Takahashi:** Conceptualization, Methodology, Writing – review & editing, Supervision.

## Declaration of Competing Interest

The authors declare that they have no known competing financial interests or personal relationships that have or could be perceived to have influenced the work reported in this article.

## Appendix Supplementary materials

Image, application 1
