# Supplementary material for: Hourly future climate scenario datasets for impact assessment of climate change considering simultaneous interactions among multiple meteorological factors
Source: Data Brief. 2022 Mar 11;42:108047. doi: 10.1016/j.dib.2022.108047 (PMC8943424; doi:10.1016/j.dib.2022.108047)
Supplement: Supplementary file 1 [file mmc1.docx]

# Appendix A. List of the folders and files

Table A shows the list of the folders and files.

**Table A. List of folder and file names.**

| **Folders** | **Files** | **Folders** | **Files** |
| --- | --- | --- | --- |
| h-CS_OR_MIROC5_rcp26 | h-CS_OR_MIROC5_rcp26_2017_01_Sapporo.csv  h-CS_OR_MIROC5_rcp26_2017_02_Sendai.csv  h-CS_OR_MIROC5_rcp26_2017_03_Tokyo.csv  h-CS_OR_MIROC5_rcp26_2017_04_Nagoya.csv  h-CS_OR_MIROC5_rcp26_2017_05_Kanazawa.csv  h-CS_OR_MIROC5_rcp26_2017_06_Osaka.csv  h-CS_OR_MIROC5_rcp26_2017_07_Hiroshima.csv  h-CS_OR_MIROC5_rcp26_2017_08_Matsuyama.csv  h-CS_OR_MIROC5_rcp26_2017_09_Fukuoka.csv  h-CS_OR_MIROC5_rcp26_2017_10_Naha.csv  h-CS_OR_MIROC5_rcp26_2018_01_Sapporo.csv  h-CS_OR_MIROC5_rcp26_2018_02_Sendai.csv  h-CS_OR_MIROC5_rcp26_2018_03_Tokyo.csv  h-CS_OR_MIROC5_rcp26_2018_04_Nagoya.csv  h-CS_OR_MIROC5_rcp26_2018_05_Kanazawa.csv  h-CS_OR_MIROC5_rcp26_2018_06_Osaka.csv  h-CS_OR_MIROC5_rcp26_2018_07_Hiroshima.csv  h-CS_OR_MIROC5_rcp26_2018_08_Matsuyama.csv  h-CS_OR_MIROC5_rcp26_2018_09_Fukuoka.csv  h-CS_OR_MIROC5_rcp26_2018_10_Naha.csv  h-CS_OR_MIROC5_rcp26_2019_01_Sapporo.csv  h-CS_OR_MIROC5_rcp26_2019_02_Sendai.csv  h-CS_OR_MIROC5_rcp26_2019_03_Tokyo.csv  h-CS_OR_MIROC5_rcp26_2019_04_Nagoya.csv  h-CS_OR_MIROC5_rcp26_2019_05_Kanazawa.csv  h-CS_OR_MIROC5_rcp26_2019_06_Osaka.csv  h-CS_OR_MIROC5_rcp26_2019_07_Hiroshima.csv  h-CS_OR_MIROC5_rcp26_2019_08_Matsuyama.csv  h-CS_OR_MIROC5_rcp26_2019_09_Fukuoka.csv  h-CS_OR_MIROC5_rcp26_2019_10_Naha.csv | h-CS_OR_MIROC5_rcp85 | h-CS_OR_MIROC5_rcp85_2017_01_Sapporo.csv  h-CS_OR_MIROC5_rcp85_2017_02_Sendai.csv  h-CS_OR_MIROC5_rcp85_2017_03_Tokyo.csv  h-CS_OR_MIROC5_rcp85_2017_04_Nagoya.csv  h-CS_OR_MIROC5_rcp85_2017_05_Kanazawa.csv  h-CS_OR_MIROC5_rcp85_2017_06_Osaka.csv  h-CS_OR_MIROC5_rcp85_2017_07_Hiroshima.csv  h-CS_OR_MIROC5_rcp85_2017_08_Matsuyama.csv  h-CS_OR_MIROC5_rcp85_2017_09_Fukuoka.csv  h-CS_OR_MIROC5_rcp85_2017_10_Naha.csv  h-CS_OR_MIROC5_rcp85_2018_01_Sapporo.csv  h-CS_OR_MIROC5_rcp85_2018_02_Sendai.csv  h-CS_OR_MIROC5_rcp85_2018_03_Tokyo.csv  h-CS_OR_MIROC5_rcp85_2018_04_Nagoya.csv  h-CS_OR_MIROC5_rcp85_2018_05_Kanazawa.csv  h-CS_OR_MIROC5_rcp85_2018_06_Osaka.csv  h-CS_OR_MIROC5_rcp85_2018_07_Hiroshima.csv  h-CS_OR_MIROC5_rcp85_2018_08_Matsuyama.csv  h-CS_OR_MIROC5_rcp85_2018_09_Fukuoka.csv  h-CS_OR_MIROC5_rcp85_2018_10_Naha.csv  h-CS_OR_MIROC5_rcp85_2019_01_Sapporo.csv  h-CS_OR_MIROC5_rcp85_2019_02_Sendai.csv  h-CS_OR_MIROC5_rcp85_2019_03_Tokyo.csv  h-CS_OR_MIROC5_rcp85_2019_04_Nagoya.csv  h-CS_OR_MIROC5_rcp85_2019_05_Kanazawa.csv  h-CS_OR_MIROC5_rcp85_2019_06_Osaka.csv  h-CS_OR_MIROC5_rcp85_2019_07_Hiroshima.csv  h-CS_OR_MIROC5_rcp85_2019_08_Matsuyama.csv  h-CS_OR_MIROC5_rcp85_2019_09_Fukuoka.csv  h-CS_OR_MIROC5_rcp85_2019_10_Naha.csv |
| h-CS_OR_MRI-CGCM3_rcp26 | h-CS_OR_MRI-CGCM3_rcp26_2017_01_Sapporo.csv  h-CS_OR_MRI-CGCM3_rcp26_2017_02_Sendai.csv  h-CS_OR_MRI-CGCM3_rcp26_2017_03_Tokyo.csv  h-CS_OR_MRI-CGCM3_rcp26_2017_04_Nagoya.csv  h-CS_OR_MRI-CGCM3_rcp26_2017_05_Kanazawa.csv  h-CS_OR_MRI-CGCM3_rcp26_2017_06_Osaka.csv  h-CS_OR_MRI-CGCM3_rcp26_2017_07_Hiroshima.csv  h-CS_OR_MRI-CGCM3_rcp26_2017_08_Matsuyama.csv  h-CS_OR_MRI-CGCM3_rcp26_2017_09_Fukuoka.csv  h-CS_OR_MRI-CGCM3_rcp26_2017_10_Naha.csv  h-CS_OR_MRI-CGCM3_rcp26_2018_01_Sapporo.csv  h-CS_OR_MRI-CGCM3_rcp26_2018_02_Sendai.csv  h-CS_OR_MRI-CGCM3_rcp26_2018_03_Tokyo.csv  h-CS_OR_MRI-CGCM3_rcp26_2018_04_Nagoya.csv  h-CS_OR_MRI-CGCM3_rcp26_2018_05_Kanazawa.csv  h-CS_OR_MRI-CGCM3_rcp26_2018_06_Osaka.csv  h-CS_OR_MRI-CGCM3_rcp26_2018_07_Hiroshima.csv  h-CS_OR_MRI-CGCM3_rcp26_2018_08_Matsuyama.csv  h-CS_OR_MRI-CGCM3_rcp26_2018_09_Fukuoka.csv  h-CS_OR_MRI-CGCM3_rcp26_2018_10_Naha.csv  h-CS_OR_MRI-CGCM3_rcp26_2019_01_Sapporo.csv  h-CS_OR_MRI-CGCM3_rcp26_2019_02_Sendai.csv  h-CS_OR_MRI-CGCM3_rcp26_2019_03_Tokyo.csv  h-CS_OR_MRI-CGCM3_rcp26_2019_04_Nagoya.csv  h-CS_OR_MRI-CGCM3_rcp26_2019_05_Kanazawa.csv  h-CS_OR_MRI-CGCM3_rcp26_2019_06_Osaka.csv  h-CS_OR_MRI-CGCM3_rcp26_2019_07_Hiroshima.csv  h-CS_OR_MRI-CGCM3_rcp26_2019_08_Matsuyama.csv  h-CS_OR_MRI-CGCM3_rcp26_2019_09_Fukuoka.csv  h-CS_OR_MRI-CGCM3_rcp26_2019_10_Naha.csv | h-CS_OR_MRI-CGCM3_rcp85 | h-CS_OR_MRI-CGCM3_rcp85_2017_01_Sapporo.csv  h-CS_OR_MRI-CGCM3_rcp85_2017_02_Sendai.csv  h-CS_OR_MRI-CGCM3_rcp85_2017_03_Tokyo.csv  h-CS_OR_MRI-CGCM3_rcp85_2017_04_Nagoya.csv  h-CS_OR_MRI-CGCM3_rcp85_2017_05_Kanazawa.csv  h-CS_OR_MRI-CGCM3_rcp85_2017_06_Osaka.csv  h-CS_OR_MRI-CGCM3_rcp85_2017_07_Hiroshima.csv  h-CS_OR_MRI-CGCM3_rcp85_2017_08_Matsuyama.csv  h-CS_OR_MRI-CGCM3_rcp85_2017_09_Fukuoka.csv  h-CS_OR_MRI-CGCM3_rcp85_2017_10_Naha.csv  h-CS_OR_MRI-CGCM3_rcp85_2018_01_Sapporo.csv  h-CS_OR_MRI-CGCM3_rcp85_2018_02_Sendai.csv  h-CS_OR_MRI-CGCM3_rcp85_2018_03_Tokyo.csv  h-CS_OR_MRI-CGCM3_rcp85_2018_04_Nagoya.csv  h-CS_OR_MRI-CGCM3_rcp85_2018_05_Kanazawa.csv  h-CS_OR_MRI-CGCM3_rcp85_2018_06_Osaka.csv  h-CS_OR_MRI-CGCM3_rcp85_2018_07_Hiroshima.csv  h-CS_OR_MRI-CGCM3_rcp85_2018_08_Matsuyama.csv  h-CS_OR_MRI-CGCM3_rcp85_2018_09_Fukuoka.csv  h-CS_OR_MRI-CGCM3_rcp85_2018_10_Naha.csv  h-CS_OR_MRI-CGCM3_rcp85_2019_01_Sapporo.csv  h-CS_OR_MRI-CGCM3_rcp85_2019_02_Sendai.csv  h-CS_OR_MRI-CGCM3_rcp85_2019_03_Tokyo.csv  h-CS_OR_MRI-CGCM3_rcp85_2019_04_Nagoya.csv  h-CS_OR_MRI-CGCM3_rcp85_2019_05_Kanazawa.csv  h-CS_OR_MRI-CGCM3_rcp85_2019_06_Osaka.csv  h-CS_OR_MRI-CGCM3_rcp85_2019_07_Hiroshima.csv  h-CS_OR_MRI-CGCM3_rcp85_2019_08_Matsuyama.csv  h-CS_OR_MRI-CGCM3_rcp85_2019_09_Fukuoka.csv  h-CS_OR_MRI-CGCM3_rcp85_2019_10_Naha.csv |
| h-CS_OR_GFDL CM3_rcp26 | h-CS_OR_GFDL CM3_rcp26_2017_01_Sapporo.csv  h-CS_OR_GFDL CM3_rcp26_2017_02_Sendai.csv  h-CS_OR_GFDL CM3_rcp26_2017_03_Tokyo.csv  h-CS_OR_GFDL CM3_rcp26_2017_04_Nagoya.csv  h-CS_OR_GFDL CM3_rcp26_2017_05_Kanazawa.csv  h-CS_OR_GFDL CM3_rcp26_2017_06_Osaka.csv  h-CS_OR_GFDL CM3_rcp26_2017_07_Hiroshima.csv  h-CS_OR_GFDL CM3_rcp26_2017_08_Matsuyama.csv  h-CS_OR_GFDL CM3_rcp26_2017_09_Fukuoka.csv  h-CS_OR_GFDL CM3_rcp26_2017_10_Naha.csv  h-CS_OR_GFDL CM3_rcp26_2018_01_Sapporo.csv  h-CS_OR_GFDL CM3_rcp26_2018_02_Sendai.csv  h-CS_OR_GFDL CM3_rcp26_2018_03_Tokyo.csv  h-CS_OR_GFDL CM3_rcp26_2018_04_Nagoya.csv  h-CS_OR_GFDL CM3_rcp26_2018_05_Kanazawa.csv  h-CS_OR_GFDL CM3_rcp26_2018_06_Osaka.csv  h-CS_OR_GFDL CM3_rcp26_2018_07_Hiroshima.csv  h-CS_OR_GFDL CM3_rcp26_2018_08_Matsuyama.csv  h-CS_OR_GFDL CM3_rcp26_2018_09_Fukuoka.csv  h-CS_OR_GFDL CM3_rcp26_2018_10_Naha.csv  h-CS_OR_GFDL CM3_rcp26_2019_01_Sapporo.csv  h-CS_OR_GFDL CM3_rcp26_2019_02_Sendai.csv  h-CS_OR_GFDL CM3_rcp26_2019_03_Tokyo.csv  h-CS_OR_GFDL CM3_rcp26_2019_04_Nagoya.csv  h-CS_OR_GFDL CM3_rcp26_2019_05_Kanazawa.csv  h-CS_OR_GFDL CM3_rcp26_2019_06_Osaka.csv  h-CS_OR_GFDL CM3_rcp26_2019_07_Hiroshima.csv  h-CS_OR_GFDL CM3_rcp26_2019_08_Matsuyama.csv  h-CS_OR_GFDL CM3_rcp26_2019_09_Fukuoka.csv  h-CS_OR_GFDL CM3_rcp26_2019_10_Naha.csv | h-CS_OR_GFDL CM3_rcp85 | h-CS_OR_GFDL CM3_rcp85_2017_01_Sapporo.csv  h-CS_OR_GFDL CM3_rcp85_2017_02_Sendai.csv  h-CS_OR_GFDL CM3_rcp85_2017_03_Tokyo.csv  h-CS_OR_GFDL CM3_rcp85_2017_04_Nagoya.csv  h-CS_OR_GFDL CM3_rcp85_2017_05_Kanazawa.csv  h-CS_OR_GFDL CM3_rcp85_2017_06_Osaka.csv  h-CS_OR_GFDL CM3_rcp85_2017_07_Hiroshima.csv  h-CS_OR_GFDL CM3_rcp85_2017_08_Matsuyama.csv  h-CS_OR_GFDL CM3_rcp85_2017_09_Fukuoka.csv  h-CS_OR_GFDL CM3_rcp85_2017_10_Naha.csv  h-CS_OR_GFDL CM3_rcp85_2018_01_Sapporo.csv  h-CS_OR_GFDL CM3_rcp85_2018_02_Sendai.csv  h-CS_OR_GFDL CM3_rcp85_2018_03_Tokyo.csv  h-CS_OR_GFDL CM3_rcp85_2018_04_Nagoya.csv  h-CS_OR_GFDL CM3_rcp85_2018_05_Kanazawa.csv  h-CS_OR_GFDL CM3_rcp85_2018_06_Osaka.csv  h-CS_OR_GFDL CM3_rcp85_2018_07_Hiroshima.csv  h-CS_OR_GFDL CM3_rcp85_2018_08_Matsuyama.csv  h-CS_OR_GFDL CM3_rcp85_2018_09_Fukuoka.csv  h-CS_OR_GFDL CM3_rcp85_2018_10_Naha.csv  h-CS_OR_GFDL CM3_rcp85_2019_01_Sapporo.csv  h-CS_OR_GFDL CM3_rcp85_2019_02_Sendai.csv  h-CS_OR_GFDL CM3_rcp85_2019_03_Tokyo.csv  h-CS_OR_GFDL CM3_rcp85_2019_04_Nagoya.csv  h-CS_OR_GFDL CM3_rcp85_2019_05_Kanazawa.csv  h-CS_OR_GFDL CM3_rcp85_2019_06_Osaka.csv  h-CS_OR_GFDL CM3_rcp85_2019_07_Hiroshima.csv  h-CS_OR_GFDL CM3_rcp85_2019_08_Matsuyama.csv  h-CS_OR_GFDL CM3_rcp85_2019_09_Fukuoka.csv  h-CS_OR_GFDL CM3_rcp85_2019_10_Naha.csv |
| h-CS_OR_HADGEM2-ES_rcp26 | h-CS_OR_HADGEM2-ES_rcp26_2017_01_Sapporo.csv  h-CS_OR_HADGEM2-ES_rcp26_2017_02_Sendai.csv  h-CS_OR_HADGEM2-ES_rcp26_2017_03_Tokyo.csv  h-CS_OR_HADGEM2-ES_rcp26_2017_04_Nagoya.csv  h-CS_OR_HADGEM2-ES_rcp26_2017_05_Kanazawa.csv  h-CS_OR_HADGEM2-ES_rcp26_2017_06_Osaka.csv  h-CS_OR_HADGEM2-ES_rcp26_2017_07_Hiroshima.csv  h-CS_OR_HADGEM2-ES_rcp26_2017_08_Matsuyama.csv  h-CS_OR_HADGEM2-ES_rcp26_2017_09_Fukuoka.csv  h-CS_OR_HADGEM2-ES_rcp26_2017_10_Naha.csv  h-CS_OR_HADGEM2-ES_rcp26_2018_01_Sapporo.csv  h-CS_OR_HADGEM2-ES_rcp26_2018_02_Sendai.csv  h-CS_OR_HADGEM2-ES_rcp26_2018_03_Tokyo.csv  h-CS_OR_HADGEM2-ES_rcp26_2018_04_Nagoya.csv  h-CS_OR_HADGEM2-ES_rcp26_2018_05_Kanazawa.csv  h-CS_OR_HADGEM2-ES_rcp26_2018_06_Osaka.csv  h-CS_OR_HADGEM2-ES_rcp26_2018_07_Hiroshima.csv  h-CS_OR_HADGEM2-ES_rcp26_2018_08_Matsuyama.csv  h-CS_OR_HADGEM2-ES_rcp26_2018_09_Fukuoka.csv  h-CS_OR_HADGEM2-ES_rcp26_2018_10_Naha.csv  h-CS_OR_HADGEM2-ES_rcp26_2019_01_Sapporo.csv  h-CS_OR_HADGEM2-ES_rcp26_2019_02_Sendai.csv  h-CS_OR_HADGEM2-ES_rcp26_2019_03_Tokyo.csv  h-CS_OR_HADGEM2-ES_rcp26_2019_04_Nagoya.csv  h-CS_OR_HADGEM2-ES_rcp26_2019_05_Kanazawa.csv  h-CS_OR_HADGEM2-ES_rcp26_2019_06_Osaka.csv  h-CS_OR_HADGEM2-ES_rcp26_2019_07_Hiroshima.csv  h-CS_OR_HADGEM2-ES_rcp26_2019_08_Matsuyama.csv  h-CS_OR_HADGEM2-ES_rcp26_2019_09_Fukuoka.csv  h-CS_OR_HADGEM2-ES_rcp26_2019_10_Naha.csv | h-CS_OR_HADGEM2-ES_rcp85 | h-CS_OR_HADGEM2-ES_rcp85_2017_01_Sapporo.csv  h-CS_OR_HADGEM2-ES_rcp85_2017_02_Sendai.csv  h-CS_OR_HADGEM2-ES_rcp85_2017_03_Tokyo.csv  h-CS_OR_HADGEM2-ES_rcp85_2017_04_Nagoya.csv  h-CS_OR_HADGEM2-ES_rcp85_2017_05_Kanazawa.csv  h-CS_OR_HADGEM2-ES_rcp85_2017_06_Osaka.csv  h-CS_OR_HADGEM2-ES_rcp85_2017_07_Hiroshima.csv  h-CS_OR_HADGEM2-ES_rcp85_2017_08_Matsuyama.csv  h-CS_OR_HADGEM2-ES_rcp85_2017_09_Fukuoka.csv  h-CS_OR_HADGEM2-ES_rcp85_2017_10_Naha.csv  h-CS_OR_HADGEM2-ES_rcp85_2018_01_Sapporo.csv  h-CS_OR_HADGEM2-ES_rcp85_2018_02_Sendai.csv  h-CS_OR_HADGEM2-ES_rcp85_2018_03_Tokyo.csv  h-CS_OR_HADGEM2-ES_rcp85_2018_04_Nagoya.csv  h-CS_OR_HADGEM2-ES_rcp85_2018_05_Kanazawa.csv  h-CS_OR_HADGEM2-ES_rcp85_2018_06_Osaka.csv  h-CS_OR_HADGEM2-ES_rcp85_2018_07_Hiroshima.csv  h-CS_OR_HADGEM2-ES_rcp85_2018_08_Matsuyama.csv  h-CS_OR_HADGEM2-ES_rcp85_2018_09_Fukuoka.csv  h-CS_OR_HADGEM2-ES_rcp85_2018_10_Naha.csv  h-CS_OR_HADGEM2-ES_rcp85_2019_01_Sapporo.csv  h-CS_OR_HADGEM2-ES_rcp85_2019_02_Sendai.csv  h-CS_OR_HADGEM2-ES_rcp85_2019_03_Tokyo.csv  h-CS_OR_HADGEM2-ES_rcp85_2019_04_Nagoya.csv  h-CS_OR_HADGEM2-ES_rcp85_2019_05_Kanazawa.csv  h-CS_OR_HADGEM2-ES_rcp85_2019_06_Osaka.csv  h-CS_OR_HADGEM2-ES_rcp85_2019_07_Hiroshima.csv  h-CS_OR_HADGEM2-ES_rcp85_2019_08_Matsuyama.csv  h-CS_OR_HADGEM2-ES_rcp85_2019_09_Fukuoka.csv  h-CS_OR_HADGEM2-ES_rcp85_2019_10_Naha.csv |
| h-CS_CN_MIROC5_rcp26 | h-CS_CN_MIROC5_rcp26_2017_01_Sapporo.csv  h-CS_CN_MIROC5_rcp26_2017_02_Sendai.csv  h-CS_CN_MIROC5_rcp26_2017_03_Tokyo.csv  h-CS_CN_MIROC5_rcp26_2017_04_Nagoya.csv  h-CS_CN_MIROC5_rcp26_2017_05_Kanazawa.csv  h-CS_CN_MIROC5_rcp26_2017_06_Osaka.csv  h-CS_CN_MIROC5_rcp26_2017_07_Hiroshima.csv  h-CS_CN_MIROC5_rcp26_2017_08_Matsuyama.csv  h-CS_CN_MIROC5_rcp26_2017_09_Fukuoka.csv  h-CS_CN_MIROC5_rcp26_2017_10_Naha.csv  h-CS_CN_MIROC5_rcp26_2018_01_Sapporo.csv  h-CS_CN_MIROC5_rcp26_2018_02_Sendai.csv  h-CS_CN_MIROC5_rcp26_2018_03_Tokyo.csv  h-CS_CN_MIROC5_rcp26_2018_04_Nagoya.csv  h-CS_CN_MIROC5_rcp26_2018_05_Kanazawa.csv  h-CS_CN_MIROC5_rcp26_2018_06_Osaka.csv  h-CS_CN_MIROC5_rcp26_2018_07_Hiroshima.csv  h-CS_CN_MIROC5_rcp26_2018_08_Matsuyama.csv  h-CS_CN_MIROC5_rcp26_2018_09_Fukuoka.csv  h-CS_CN_MIROC5_rcp26_2018_10_Naha.csv  h-CS_CN_MIROC5_rcp26_2019_01_Sapporo.csv  h-CS_CN_MIROC5_rcp26_2019_02_Sendai.csv  h-CS_CN_MIROC5_rcp26_2019_03_Tokyo.csv  h-CS_CN_MIROC5_rcp26_2019_04_Nagoya.csv  h-CS_CN_MIROC5_rcp26_2019_05_Kanazawa.csv  h-CS_CN_MIROC5_rcp26_2019_06_Osaka.csv  h-CS_CN_MIROC5_rcp26_2019_07_Hiroshima.csv  h-CS_CN_MIROC5_rcp26_2019_08_Matsuyama.csv  h-CS_CN_MIROC5_rcp26_2019_09_Fukuoka.csv  h-CS_CN_MIROC5_rcp26_2019_10_Naha.csv | h-CS_CN_MIROC5_rcp85 | h-CS_CN_MIROC5_rcp85_2017_01_Sapporo.csv  h-CS_CN_MIROC5_rcp85_2017_02_Sendai.csv  h-CS_CN_MIROC5_rcp85_2017_03_Tokyo.csv  h-CS_CN_MIROC5_rcp85_2017_04_Nagoya.csv  h-CS_CN_MIROC5_rcp85_2017_05_Kanazawa.csv  h-CS_CN_MIROC5_rcp85_2017_06_Osaka.csv  h-CS_CN_MIROC5_rcp85_2017_07_Hiroshima.csv  h-CS_CN_MIROC5_rcp85_2017_08_Matsuyama.csv  h-CS_CN_MIROC5_rcp85_2017_09_Fukuoka.csv  h-CS_CN_MIROC5_rcp85_2017_10_Naha.csv  h-CS_CN_MIROC5_rcp85_2018_01_Sapporo.csv  h-CS_CN_MIROC5_rcp85_2018_02_Sendai.csv  h-CS_CN_MIROC5_rcp85_2018_03_Tokyo.csv  h-CS_CN_MIROC5_rcp85_2018_04_Nagoya.csv  h-CS_CN_MIROC5_rcp85_2018_05_Kanazawa.csv  h-CS_CN_MIROC5_rcp85_2018_06_Osaka.csv  h-CS_CN_MIROC5_rcp85_2018_07_Hiroshima.csv  h-CS_CN_MIROC5_rcp85_2018_08_Matsuyama.csv  h-CS_CN_MIROC5_rcp85_2018_09_Fukuoka.csv  h-CS_CN_MIROC5_rcp85_2018_10_Naha.csv  h-CS_CN_MIROC5_rcp85_2019_01_Sapporo.csv  h-CS_CN_MIROC5_rcp85_2019_02_Sendai.csv  h-CS_CN_MIROC5_rcp85_2019_03_Tokyo.csv  h-CS_CN_MIROC5_rcp85_2019_04_Nagoya.csv  h-CS_CN_MIROC5_rcp85_2019_05_Kanazawa.csv  h-CS_CN_MIROC5_rcp85_2019_06_Osaka.csv  h-CS_CN_MIROC5_rcp85_2019_07_Hiroshima.csv  h-CS_CN_MIROC5_rcp85_2019_08_Matsuyama.csv  h-CS_CN_MIROC5_rcp85_2019_09_Fukuoka.csv  h-CS_CN_MIROC5_rcp85_2019_10_Naha.csv |
| h-CS_CN_MRI-CGCM3_rcp26 | h-CS_CN_MRI-CGCM3_rcp26_2017_01_Sapporo.csv  h-CS_CN_MRI-CGCM3_rcp26_2017_02_Sendai.csv  h-CS_CN_MRI-CGCM3_rcp26_2017_03_Tokyo.csv  h-CS_CN_MRI-CGCM3_rcp26_2017_04_Nagoya.csv  h-CS_CN_MRI-CGCM3_rcp26_2017_05_Kanazawa.csv  h-CS_CN_MRI-CGCM3_rcp26_2017_06_Osaka.csv  h-CS_CN_MRI-CGCM3_rcp26_2017_07_Hiroshima.csv  h-CS_CN_MRI-CGCM3_rcp26_2017_08_Matsuyama.csv  h-CS_CN_MRI-CGCM3_rcp26_2017_09_Fukuoka.csv  h-CS_CN_MRI-CGCM3_rcp26_2017_10_Naha.csv  h-CS_CN_MRI-CGCM3_rcp26_2018_01_Sapporo.csv  h-CS_CN_MRI-CGCM3_rcp26_2018_02_Sendai.csv  h-CS_CN_MRI-CGCM3_rcp26_2018_03_Tokyo.csv  h-CS_CN_MRI-CGCM3_rcp26_2018_04_Nagoya.csv  h-CS_CN_MRI-CGCM3_rcp26_2018_05_Kanazawa.csv  h-CS_CN_MRI-CGCM3_rcp26_2018_06_Osaka.csv  h-CS_CN_MRI-CGCM3_rcp26_2018_07_Hiroshima.csv  h-CS_CN_MRI-CGCM3_rcp26_2018_08_Matsuyama.csv  h-CS_CN_MRI-CGCM3_rcp26_2018_09_Fukuoka.csv  h-CS_CN_MRI-CGCM3_rcp26_2018_10_Naha.csv  h-CS_CN_MRI-CGCM3_rcp26_2019_01_Sapporo.csv  h-CS_CN_MRI-CGCM3_rcp26_2019_02_Sendai.csv  h-CS_CN_MRI-CGCM3_rcp26_2019_03_Tokyo.csv  h-CS_CN_MRI-CGCM3_rcp26_2019_04_Nagoya.csv  h-CS_CN_MRI-CGCM3_rcp26_2019_05_Kanazawa.csv  h-CS_CN_MRI-CGCM3_rcp26_2019_06_Osaka.csv  h-CS_CN_MRI-CGCM3_rcp26_2019_07_Hiroshima.csv  h-CS_CN_MRI-CGCM3_rcp26_2019_08_Matsuyama.csv  h-CS_CN_MRI-CGCM3_rcp26_2019_09_Fukuoka.csv  h-CS_CN_MRI-CGCM3_rcp26_2019_10_Naha.csv | h-CS_CN_MRI-CGCM3_rcp85 | h-CS_CN_MRI-CGCM3_rcp85_2017_01_Sapporo.csv  h-CS_CN_MRI-CGCM3_rcp85_2017_02_Sendai.csv  h-CS_CN_MRI-CGCM3_rcp85_2017_03_Tokyo.csv  h-CS_CN_MRI-CGCM3_rcp85_2017_04_Nagoya.csv  h-CS_CN_MRI-CGCM3_rcp85_2017_05_Kanazawa.csv  h-CS_CN_MRI-CGCM3_rcp85_2017_06_Osaka.csv  h-CS_CN_MRI-CGCM3_rcp85_2017_07_Hiroshima.csv  h-CS_CN_MRI-CGCM3_rcp85_2017_08_Matsuyama.csv  h-CS_CN_MRI-CGCM3_rcp85_2017_09_Fukuoka.csv  h-CS_CN_MRI-CGCM3_rcp85_2017_10_Naha.csv  h-CS_CN_MRI-CGCM3_rcp85_2018_01_Sapporo.csv  h-CS_CN_MRI-CGCM3_rcp85_2018_02_Sendai.csv  h-CS_CN_MRI-CGCM3_rcp85_2018_03_Tokyo.csv  h-CS_CN_MRI-CGCM3_rcp85_2018_04_Nagoya.csv  h-CS_CN_MRI-CGCM3_rcp85_2018_05_Kanazawa.csv  h-CS_CN_MRI-CGCM3_rcp85_2018_06_Osaka.csv  h-CS_CN_MRI-CGCM3_rcp85_2018_07_Hiroshima.csv  h-CS_CN_MRI-CGCM3_rcp85_2018_08_Matsuyama.csv  h-CS_CN_MRI-CGCM3_rcp85_2018_09_Fukuoka.csv  h-CS_CN_MRI-CGCM3_rcp85_2018_10_Naha.csv  h-CS_CN_MRI-CGCM3_rcp85_2019_01_Sapporo.csv  h-CS_CN_MRI-CGCM3_rcp85_2019_02_Sendai.csv  h-CS_CN_MRI-CGCM3_rcp85_2019_03_Tokyo.csv  h-CS_CN_MRI-CGCM3_rcp85_2019_04_Nagoya.csv  h-CS_CN_MRI-CGCM3_rcp85_2019_05_Kanazawa.csv  h-CS_CN_MRI-CGCM3_rcp85_2019_06_Osaka.csv  h-CS_CN_MRI-CGCM3_rcp85_2019_07_Hiroshima.csv  h-CS_CN_MRI-CGCM3_rcp85_2019_08_Matsuyama.csv  h-CS_CN_MRI-CGCM3_rcp85_2019_09_Fukuoka.csv  h-CS_CN_MRI-CGCM3_rcp85_2019_10_Naha.csv |
| h-CS_CN_GFDL CM3_rcp26 | h-CS_CN_GFDL CM3_rcp26_2017_01_Sapporo.csv  h-CS_CN_GFDL CM3_rcp26_2017_02_Sendai.csv  h-CS_CN_GFDL CM3_rcp26_2017_03_Tokyo.csv  h-CS_CN_GFDL CM3_rcp26_2017_04_Nagoya.csv  h-CS_CN_GFDL CM3_rcp26_2017_05_Kanazawa.csv  h-CS_CN_GFDL CM3_rcp26_2017_06_Osaka.csv  h-CS_CN_GFDL CM3_rcp26_2017_07_Hiroshima.csv  h-CS_CN_GFDL CM3_rcp26_2017_08_Matsuyama.csv  h-CS_CN_GFDL CM3_rcp26_2017_09_Fukuoka.csv  h-CS_CN_GFDL CM3_rcp26_2017_10_Naha.csv  h-CS_CN_GFDL CM3_rcp26_2018_01_Sapporo.csv  h-CS_CN_GFDL CM3_rcp26_2018_02_Sendai.csv  h-CS_CN_GFDL CM3_rcp26_2018_03_Tokyo.csv  h-CS_CN_GFDL CM3_rcp26_2018_04_Nagoya.csv  h-CS_CN_GFDL CM3_rcp26_2018_05_Kanazawa.csv  h-CS_CN_GFDL CM3_rcp26_2018_06_Osaka.csv  h-CS_CN_GFDL CM3_rcp26_2018_07_Hiroshima.csv  h-CS_CN_GFDL CM3_rcp26_2018_08_Matsuyama.csv  h-CS_CN_GFDL CM3_rcp26_2018_09_Fukuoka.csv  h-CS_CN_GFDL CM3_rcp26_2018_10_Naha.csv  h-CS_CN_GFDL CM3_rcp26_2019_01_Sapporo.csv  h-CS_CN_GFDL CM3_rcp26_2019_02_Sendai.csv  h-CS_CN_GFDL CM3_rcp26_2019_03_Tokyo.csv  h-CS_CN_GFDL CM3_rcp26_2019_04_Nagoya.csv  h-CS_CN_GFDL CM3_rcp26_2019_05_Kanazawa.csv  h-CS_CN_GFDL CM3_rcp26_2019_06_Osaka.csv  h-CS_CN_GFDL CM3_rcp26_2019_07_Hiroshima.csv  h-CS_CN_GFDL CM3_rcp26_2019_08_Matsuyama.csv  h-CS_CN_GFDL CM3_rcp26_2019_09_Fukuoka.csv  h-CS_CN_GFDL CM3_rcp26_2019_10_Naha.csv | h-CS_CN_GFDL CM3_rcp85 | h-CS_CN_GFDL CM3_rcp85_2017_01_Sapporo.csv  h-CS_CN_GFDL CM3_rcp85_2017_02_Sendai.csv  h-CS_CN_GFDL CM3_rcp85_2017_03_Tokyo.csv  h-CS_CN_GFDL CM3_rcp85_2017_04_Nagoya.csv  h-CS_CN_GFDL CM3_rcp85_2017_05_Kanazawa.csv  h-CS_CN_GFDL CM3_rcp85_2017_06_Osaka.csv  h-CS_CN_GFDL CM3_rcp85_2017_07_Hiroshima.csv  h-CS_CN_GFDL CM3_rcp85_2017_08_Matsuyama.csv  h-CS_CN_GFDL CM3_rcp85_2017_09_Fukuoka.csv  h-CS_CN_GFDL CM3_rcp85_2017_10_Naha.csv  h-CS_CN_GFDL CM3_rcp85_2018_01_Sapporo.csv  h-CS_CN_GFDL CM3_rcp85_2018_02_Sendai.csv  h-CS_CN_GFDL CM3_rcp85_2018_03_Tokyo.csv  h-CS_CN_GFDL CM3_rcp85_2018_04_Nagoya.csv  h-CS_CN_GFDL CM3_rcp85_2018_05_Kanazawa.csv  h-CS_CN_GFDL CM3_rcp85_2018_06_Osaka.csv  h-CS_CN_GFDL CM3_rcp85_2018_07_Hiroshima.csv  h-CS_CN_GFDL CM3_rcp85_2018_08_Matsuyama.csv  h-CS_CN_GFDL CM3_rcp85_2018_09_Fukuoka.csv  h-CS_CN_GFDL CM3_rcp85_2018_10_Naha.csv  h-CS_CN_GFDL CM3_rcp85_2019_01_Sapporo.csv  h-CS_CN_GFDL CM3_rcp85_2019_02_Sendai.csv  h-CS_CN_GFDL CM3_rcp85_2019_03_Tokyo.csv  h-CS_CN_GFDL CM3_rcp85_2019_04_Nagoya.csv  h-CS_CN_GFDL CM3_rcp85_2019_05_Kanazawa.csv  h-CS_CN_GFDL CM3_rcp85_2019_06_Osaka.csv  h-CS_CN_GFDL CM3_rcp85_2019_07_Hiroshima.csv  h-CS_CN_GFDL CM3_rcp85_2019_08_Matsuyama.csv  h-CS_CN_GFDL CM3_rcp85_2019_09_Fukuoka.csv  h-CS_CN_GFDL CM3_rcp85_2019_10_Naha.csv |
| h-CS_CN_HADGEM2-ES_rcp26 | h-CS_CN_HADGEM2-ES_rcp26_2017_01_Sapporo.csv  h-CS_CN_HADGEM2-ES_rcp26_2017_02_Sendai.csv  h-CS_CN_HADGEM2-ES_rcp26_2017_03_Tokyo.csv  h-CS_CN_HADGEM2-ES_rcp26_2017_04_Nagoya.csv  h-CS_CN_HADGEM2-ES_rcp26_2017_05_Kanazawa.csv  h-CS_CN_HADGEM2-ES_rcp26_2017_06_Osaka.csv  h-CS_CN_HADGEM2-ES_rcp26_2017_07_Hiroshima.csv  h-CS_CN_HADGEM2-ES_rcp26_2017_08_Matsuyama.csv  h-CS_CN_HADGEM2-ES_rcp26_2017_09_Fukuoka.csv  h-CS_CN_HADGEM2-ES_rcp26_2017_10_Naha.csv  h-CS_CN_HADGEM2-ES_rcp26_2018_01_Sapporo.csv  h-CS_CN_HADGEM2-ES_rcp26_2018_02_Sendai.csv  h-CS_CN_HADGEM2-ES_rcp26_2018_03_Tokyo.csv  h-CS_CN_HADGEM2-ES_rcp26_2018_04_Nagoya.csv  h-CS_CN_HADGEM2-ES_rcp26_2018_05_Kanazawa.csv  h-CS_CN_HADGEM2-ES_rcp26_2018_06_Osaka.csv  h-CS_CN_HADGEM2-ES_rcp26_2018_07_Hiroshima.csv  h-CS_CN_HADGEM2-ES_rcp26_2018_08_Matsuyama.csv  h-CS_CN_HADGEM2-ES_rcp26_2018_09_Fukuoka.csv  h-CS_CN_HADGEM2-ES_rcp26_2018_10_Naha.csv  h-CS_CN_HADGEM2-ES_rcp26_2019_01_Sapporo.csv  h-CS_CN_HADGEM2-ES_rcp26_2019_02_Sendai.csv  h-CS_CN_HADGEM2-ES_rcp26_2019_03_Tokyo.csv  h-CS_CN_HADGEM2-ES_rcp26_2019_04_Nagoya.csv  h-CS_CN_HADGEM2-ES_rcp26_2019_05_Kanazawa.csv  h-CS_CN_HADGEM2-ES_rcp26_2019_06_Osaka.csv  h-CS_CN_HADGEM2-ES_rcp26_2019_07_Hiroshima.csv  h-CS_CN_HADGEM2-ES_rcp26_2019_08_Matsuyama.csv  h-CS_CN_HADGEM2-ES_rcp26_2019_09_Fukuoka.csv  h-CS_CN_HADGEM2-ES_rcp26_2019_10_Naha.csv | h-CS_CN_HADGEM2-ES_rcp85 | h-CS_CN_HADGEM2-ES_rcp85_2017_01_Sapporo.csv  h-CS_CN_HADGEM2-ES_rcp85_2017_02_Sendai.csv  h-CS_CN_HADGEM2-ES_rcp85_2017_03_Tokyo.csv  h-CS_CN_HADGEM2-ES_rcp85_2017_04_Nagoya.csv  h-CS_CN_HADGEM2-ES_rcp85_2017_05_Kanazawa.csv  h-CS_CN_HADGEM2-ES_rcp85_2017_06_Osaka.csv  h-CS_CN_HADGEM2-ES_rcp85_2017_07_Hiroshima.csv  h-CS_CN_HADGEM2-ES_rcp85_2017_08_Matsuyama.csv  h-CS_CN_HADGEM2-ES_rcp85_2017_09_Fukuoka.csv  h-CS_CN_HADGEM2-ES_rcp85_2017_10_Naha.csv  h-CS_CN_HADGEM2-ES_rcp85_2018_01_Sapporo.csv  h-CS_CN_HADGEM2-ES_rcp85_2018_02_Sendai.csv  h-CS_CN_HADGEM2-ES_rcp85_2018_03_Tokyo.csv  h-CS_CN_HADGEM2-ES_rcp85_2018_04_Nagoya.csv  h-CS_CN_HADGEM2-ES_rcp85_2018_05_Kanazawa.csv  h-CS_CN_HADGEM2-ES_rcp85_2018_06_Osaka.csv  h-CS_CN_HADGEM2-ES_rcp85_2018_07_Hiroshima.csv  h-CS_CN_HADGEM2-ES_rcp85_2018_08_Matsuyama.csv  h-CS_CN_HADGEM2-ES_rcp85_2018_09_Fukuoka.csv  h-CS_CN_HADGEM2-ES_rcp85_2018_10_Naha.csv  h-CS_CN_HADGEM2-ES_rcp85_2019_01_Sapporo.csv  h-CS_CN_HADGEM2-ES_rcp85_2019_02_Sendai.csv  h-CS_CN_HADGEM2-ES_rcp85_2019_03_Tokyo.csv  h-CS_CN_HADGEM2-ES_rcp85_2019_04_Nagoya.csv  h-CS_CN_HADGEM2-ES_rcp85_2019_05_Kanazawa.csv  h-CS_CN_HADGEM2-ES_rcp85_2019_06_Osaka.csv  h-CS_CN_HADGEM2-ES_rcp85_2019_07_Hiroshima.csv  h-CS_CN_HADGEM2-ES_rcp85_2019_08_Matsuyama.csv  h-CS_CN_HADGEM2-ES_rcp85_2019_09_Fukuoka.csv  h-CS_CN_HADGEM2-ES_rcp85_2019_10_Naha.csv |

# Appendix B. Description of the scenario data


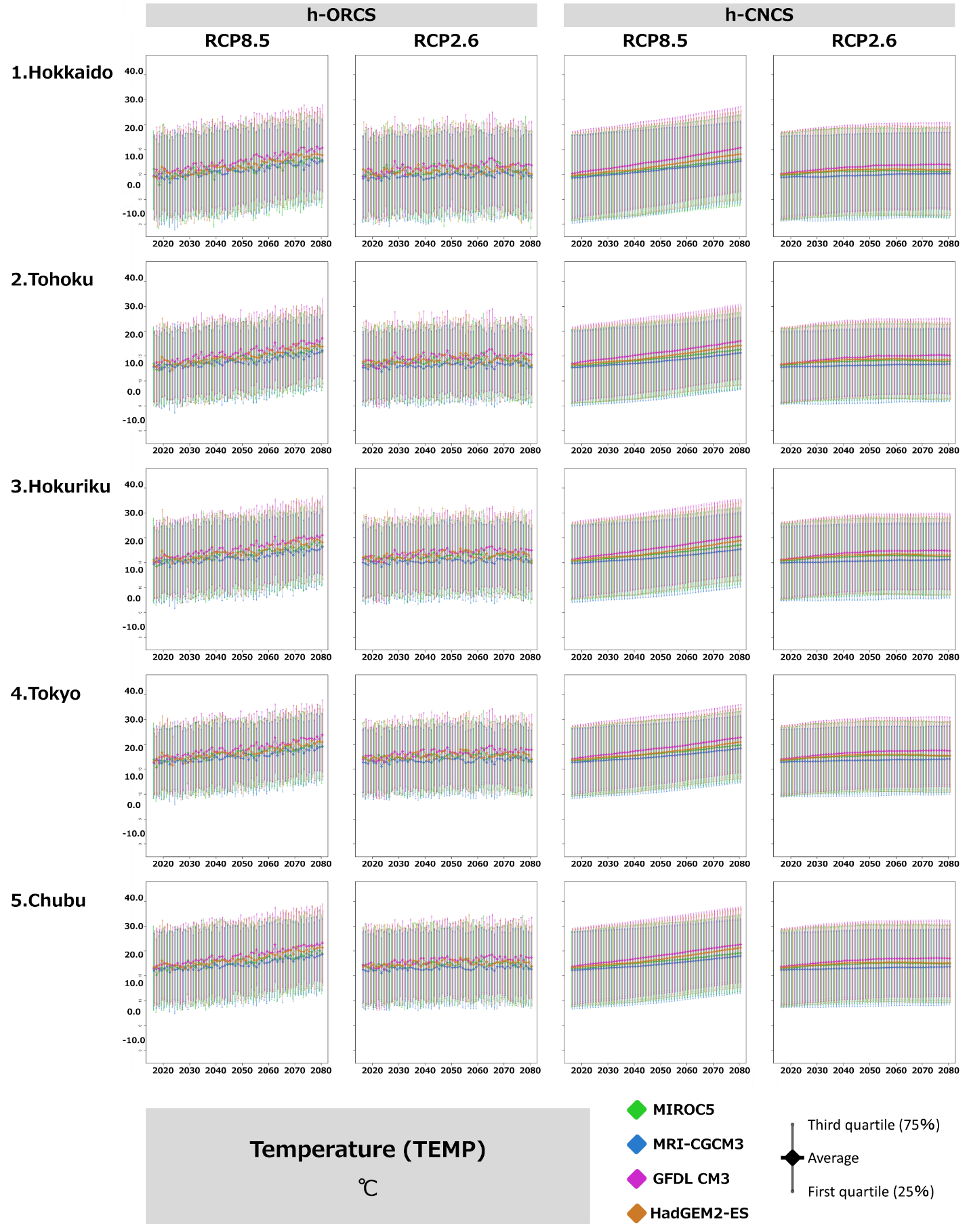


Fig. B.1. Temperature.


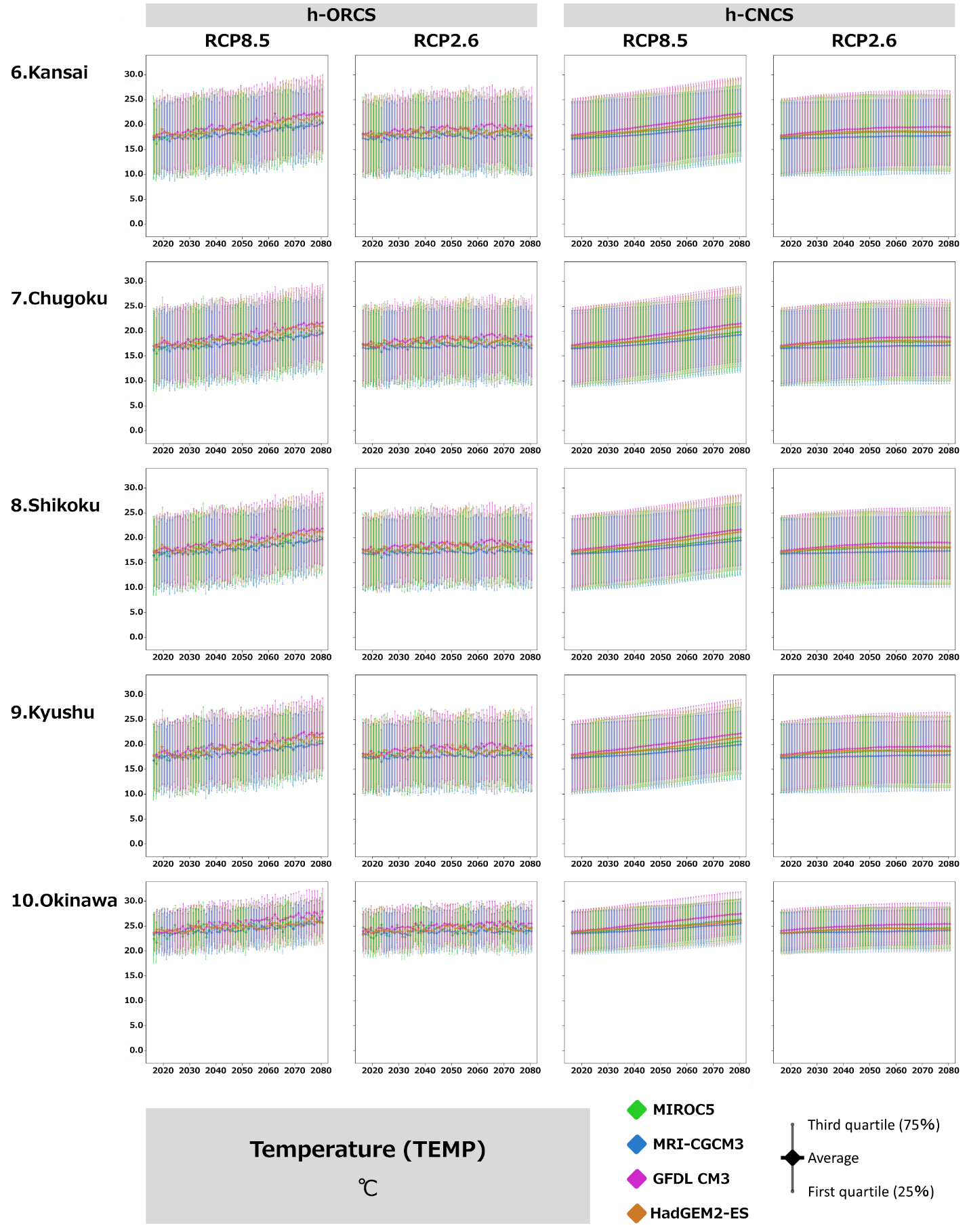


Fig. B.1. Temperature (continued).
The diamonds and vertical lines show the average and interquartile range of the temperature, respectively, for 26,280 hours (24 hours × 365 days × 3 reference years), excluding the hours on February 29.


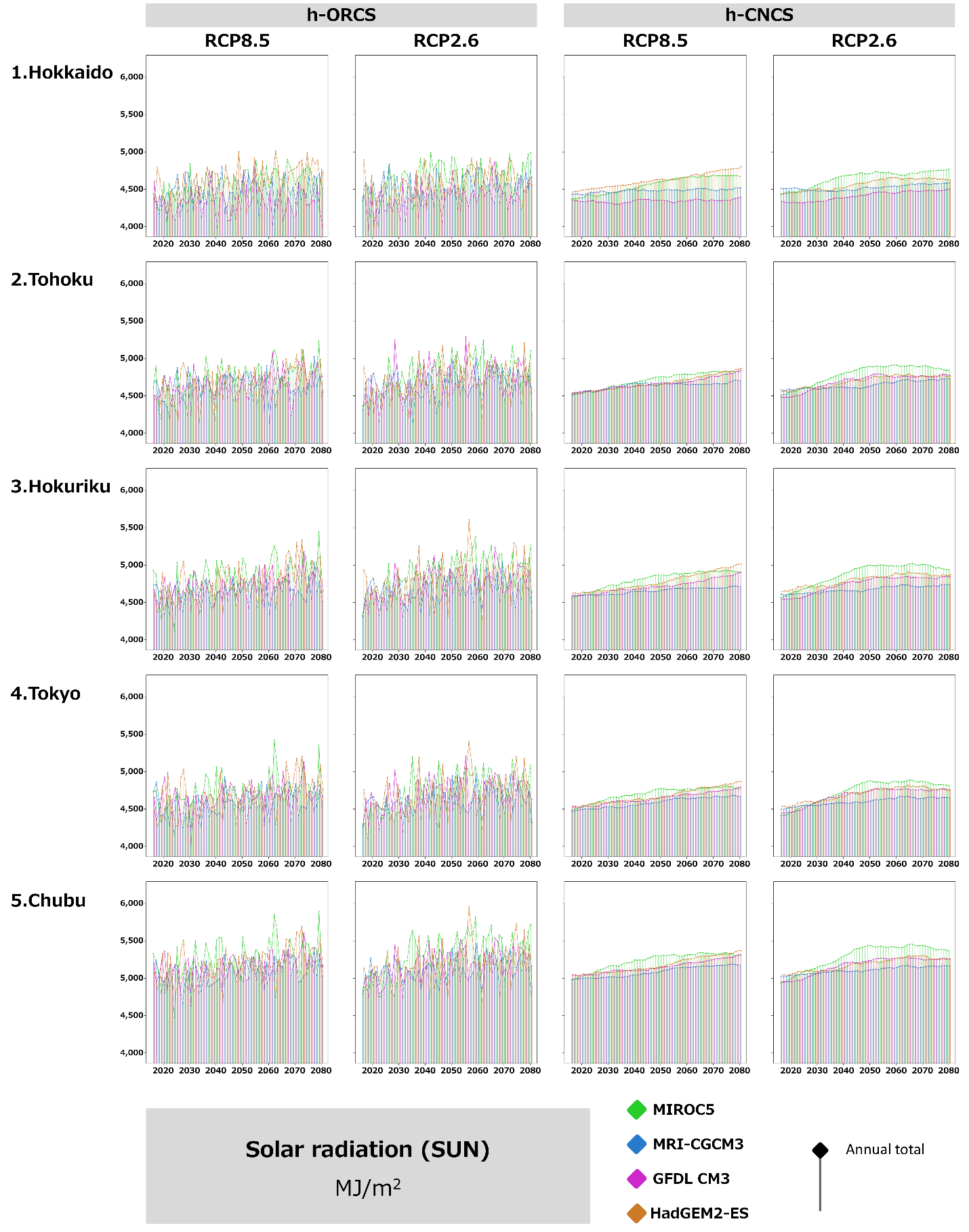


Fig. B.2. Solar radiation.


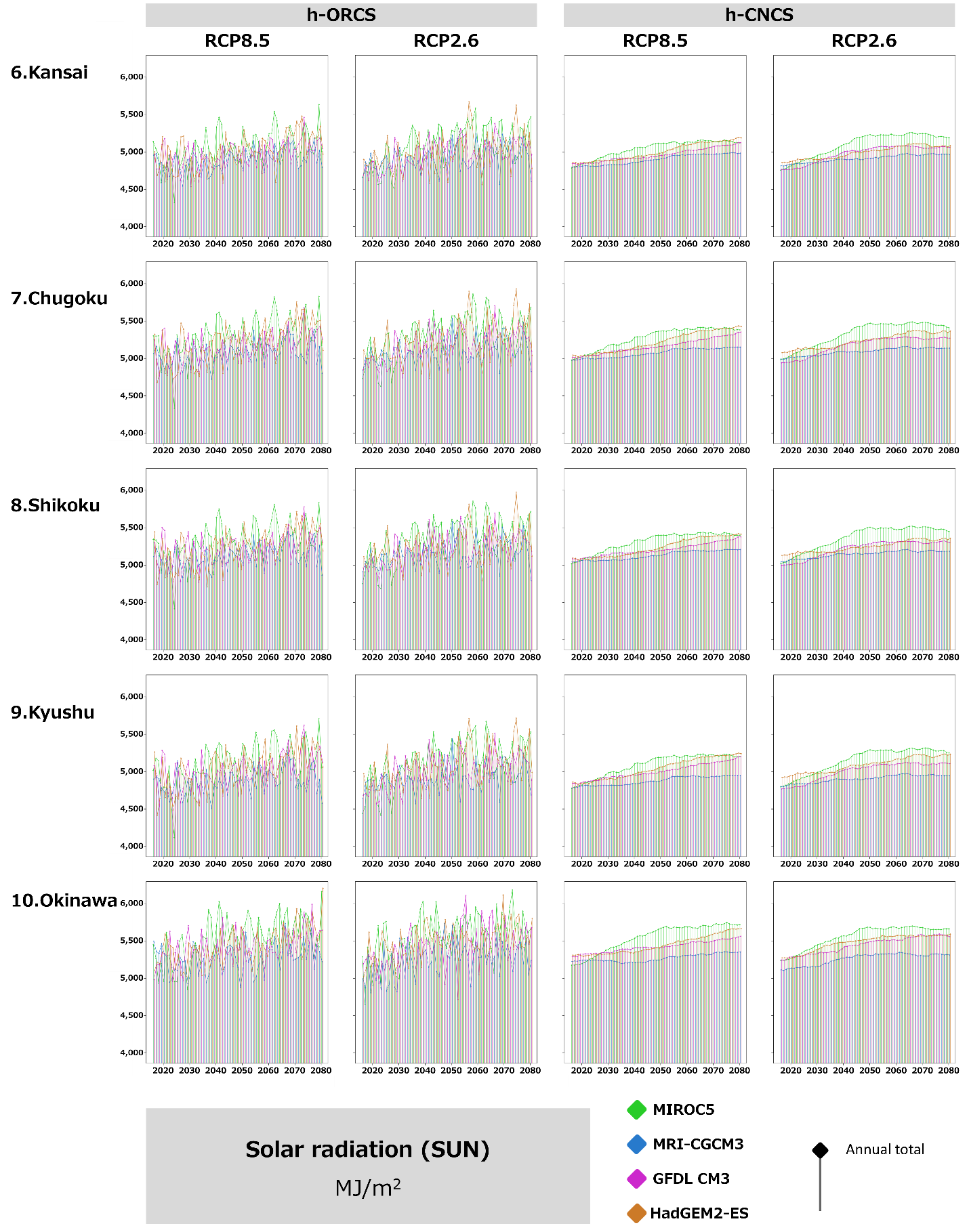


Fig. B.2. Solar radiation (continued).
Each bar shows the average annual total solar radiation; The total solar radiation values of 26,280 (24 hours × 365 days × 3 reference years) hours were divided by three.


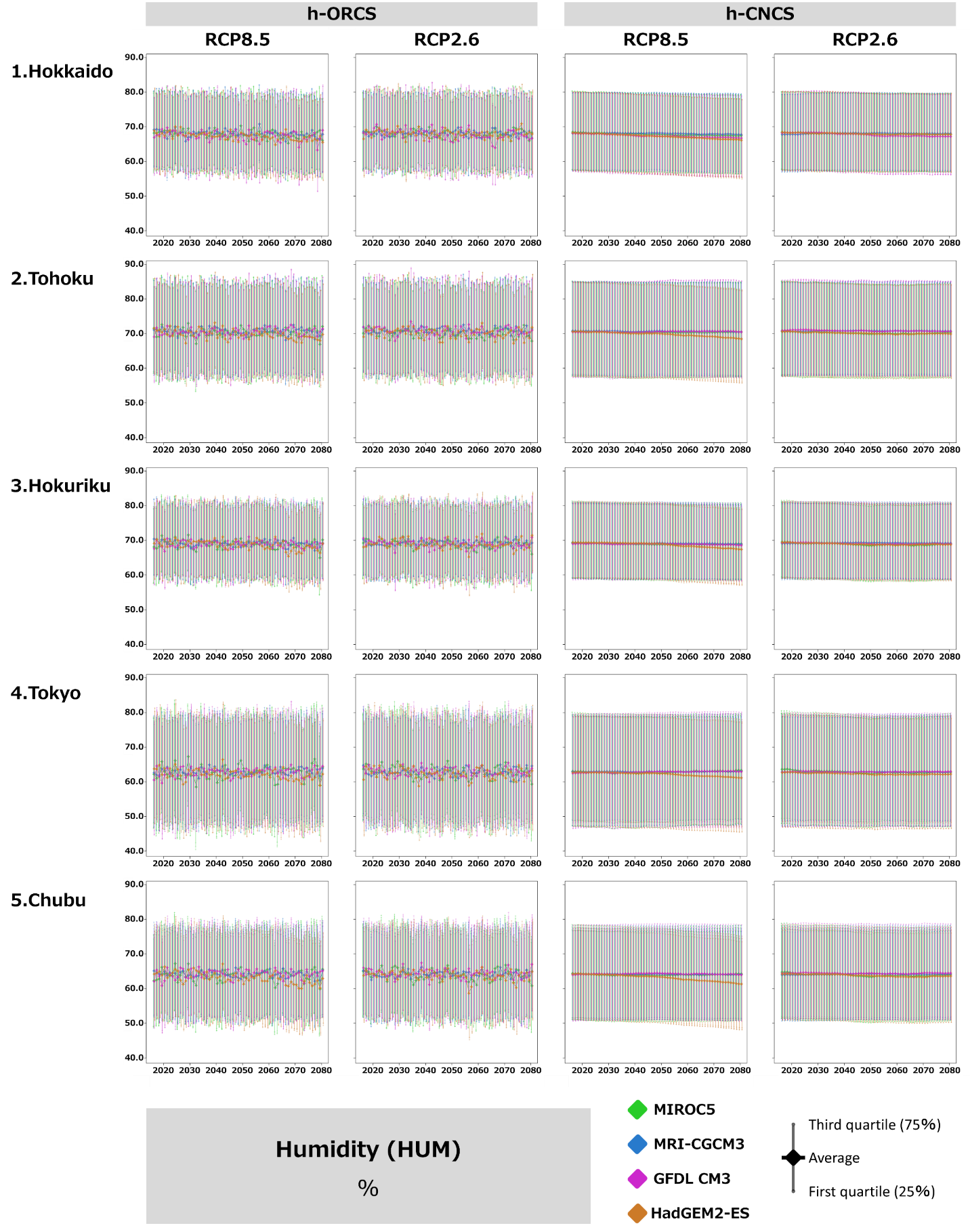


Fig. B.3. Humidity.


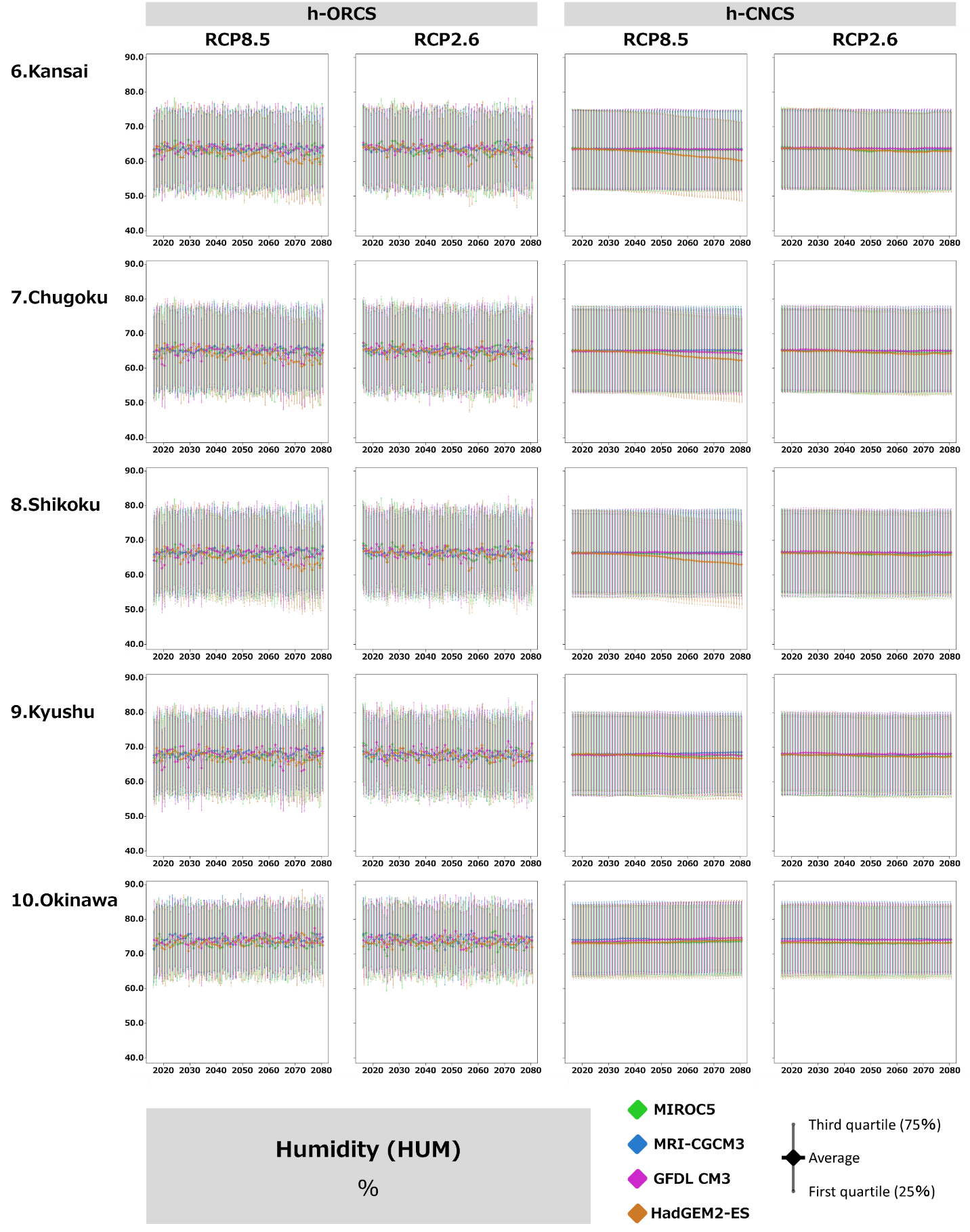


Fig. B.3. Humidity (continued).
The diamonds and vertical lines show the average and interquartile range of the relative humidity, respectively, for 26,280 hours (24 hours × 365 days × 3 reference years).


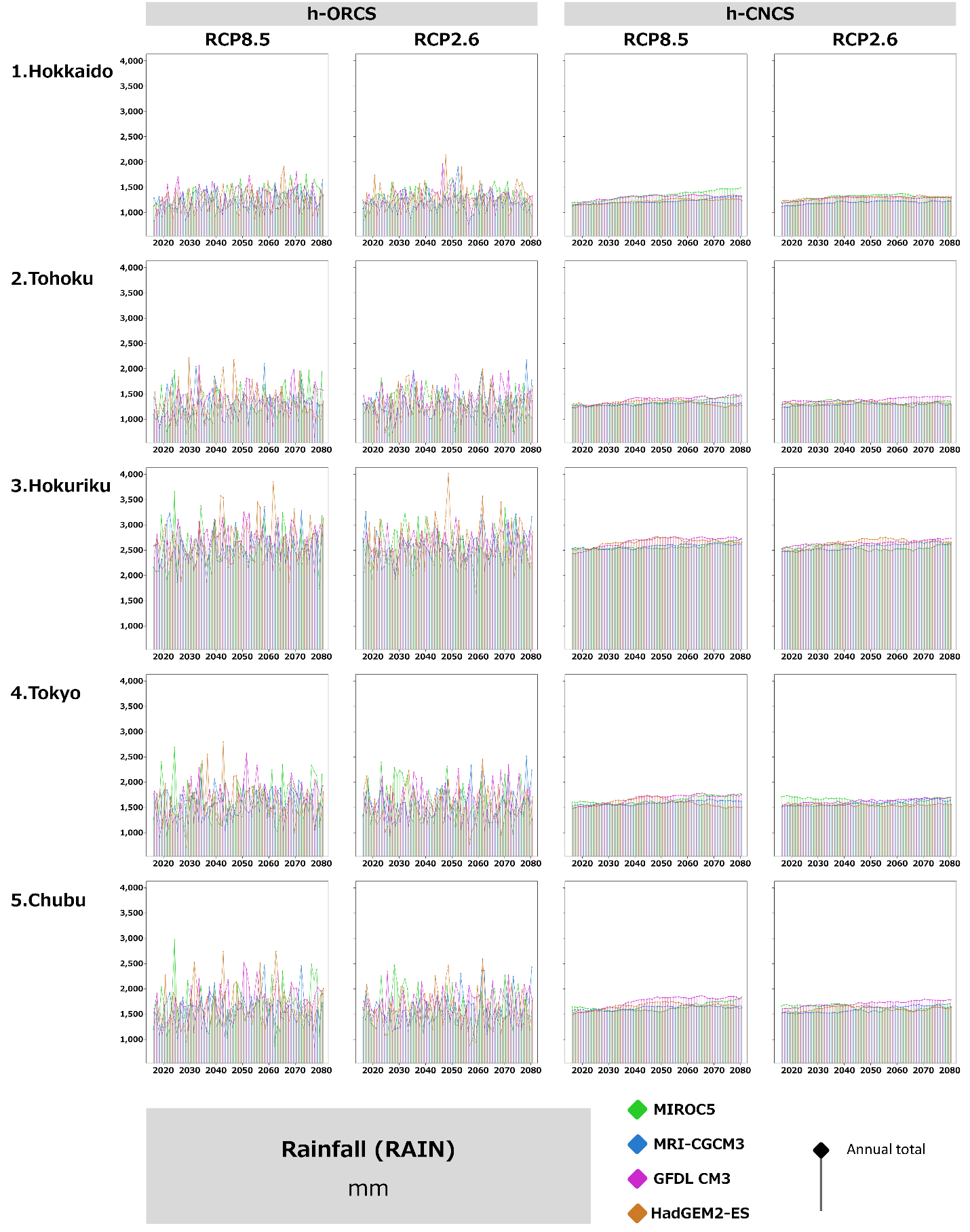


Fig. B.4. Rainfall.


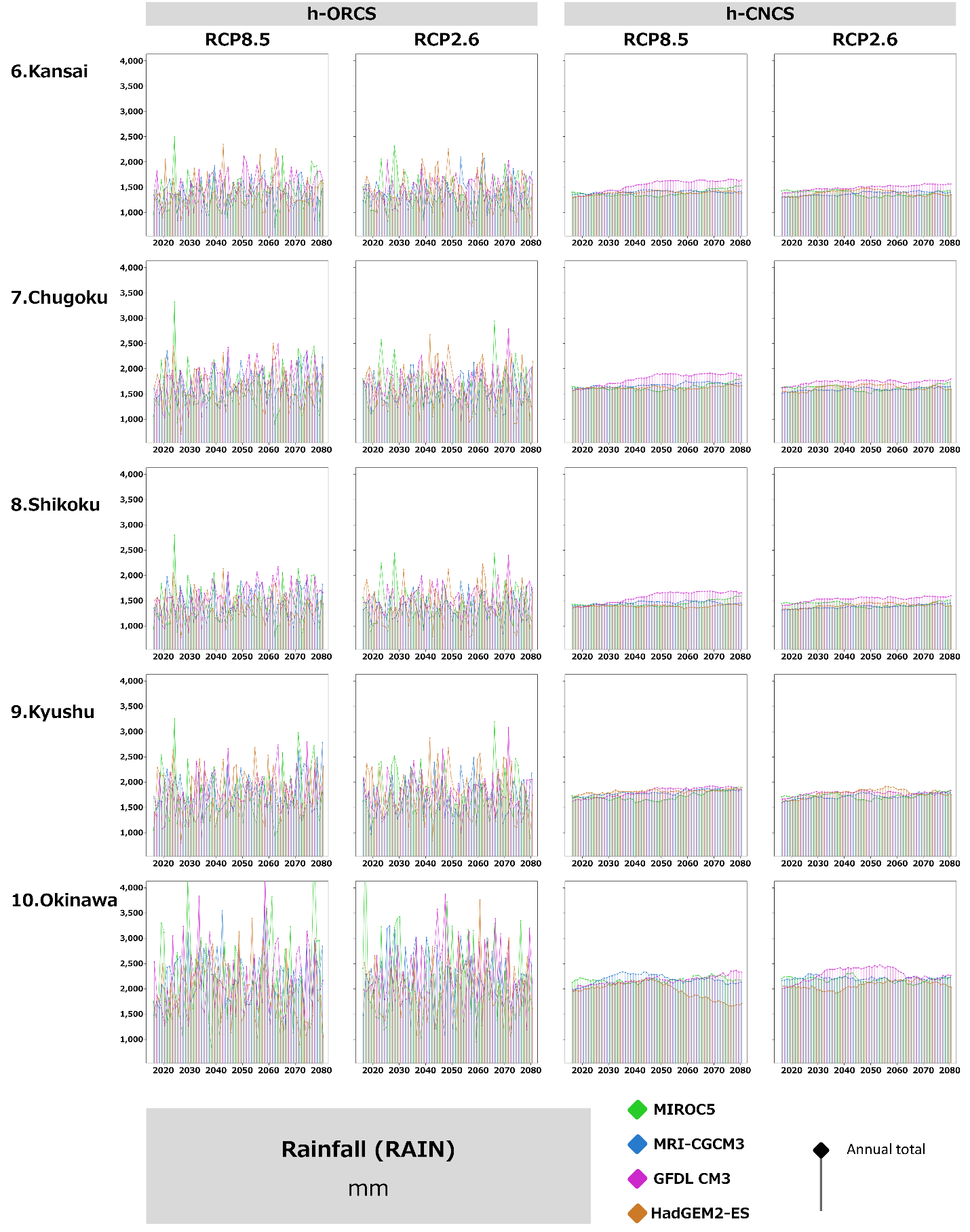


Fig. B.4. Precipitation (continued).
Each bar shows the average annual total precipitation; The total precipitation values of 26,280 (24 hours × 365 days × 3 reference years) hours were divided by three.


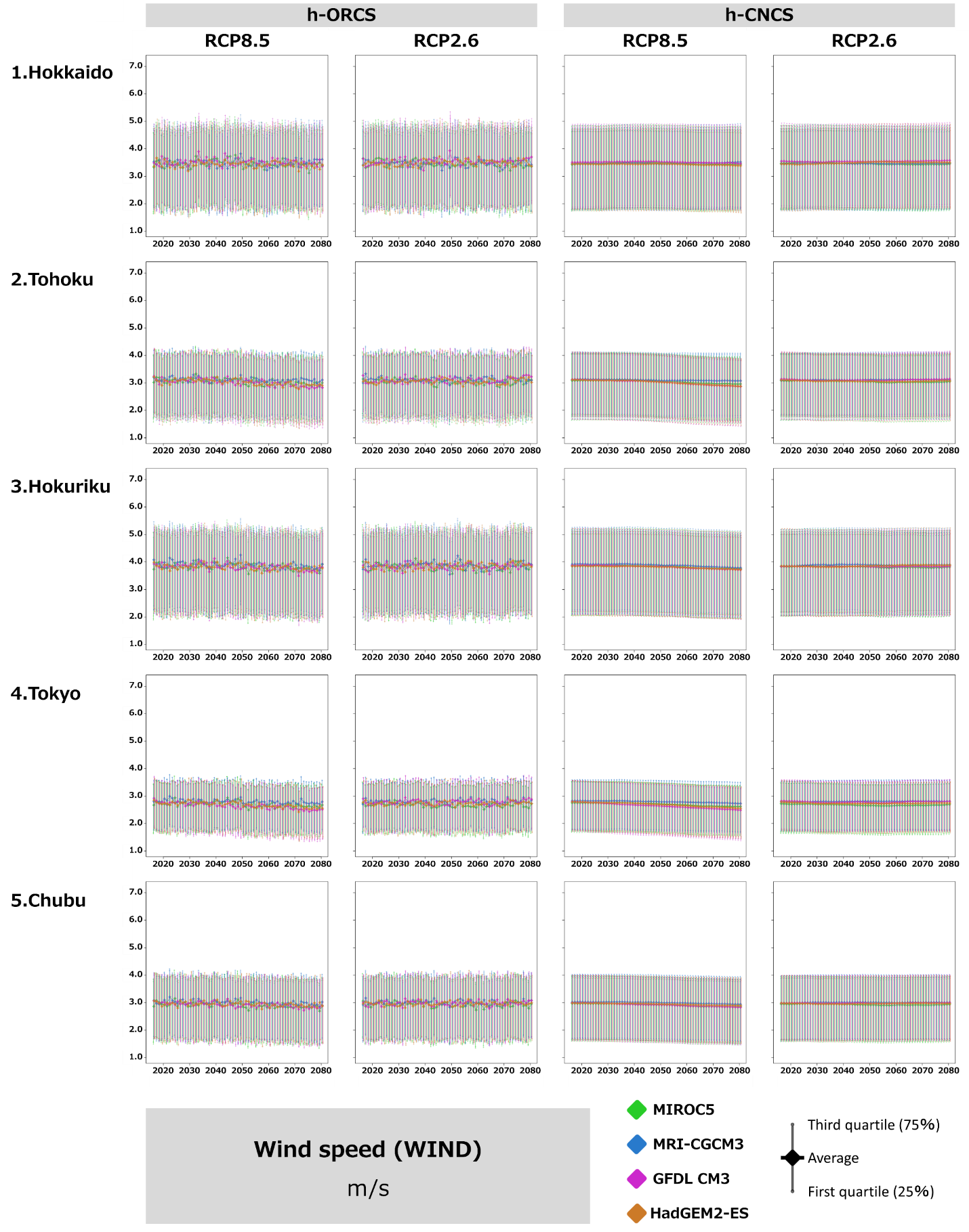


Fig. B.5. Wind speed.


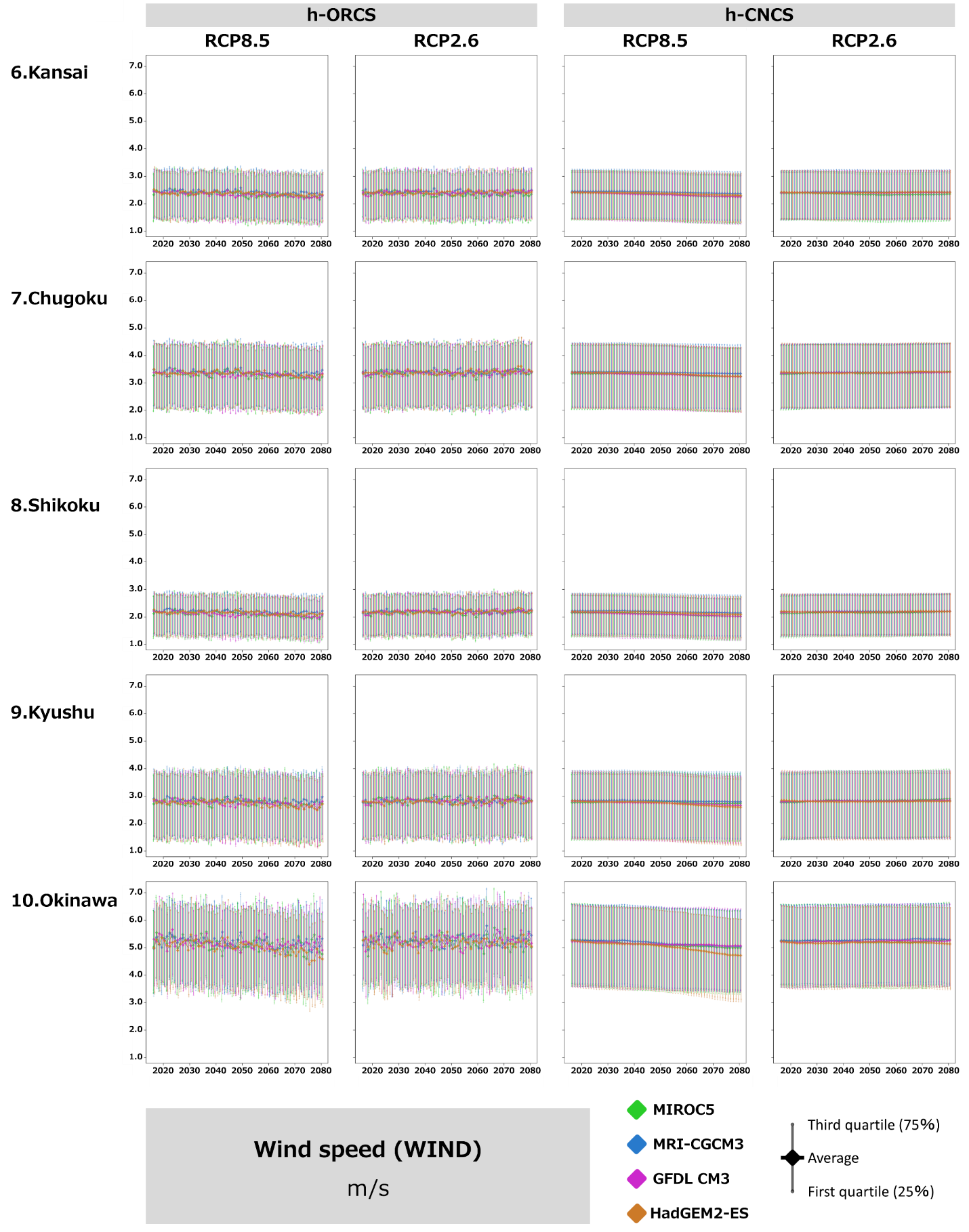


Fig. B.5. Wind speed (continued).
The diamonds and vertical lines show the average and interquartile range of the wind speed, respectively, for 26,280 hours (24 hours × 365 days × 3 reference years).
